# Biomimetic Approaches in Scaffold-Based Blood Vessel Tissue Engineering

**DOI:** 10.3390/biomimetics9070377

**Published:** 2024-06-22

**Authors:** Elisabetta Rosellini, Cristiana Giordano, Lorenzo Guidi, Maria Grazia Cascone

**Affiliations:** Department of Civil and Industrial Engineering, University of Pisa, Largo Lucio Lazzarino 1, 56122 Pisa, Italy; cristiana.giordano@phd.unipi.it (C.G.); lorenzo.guidi@phd.unipi.it (L.G.)

**Keywords:** biomimicry, natural polymers, three-layered blood vessel, functionalisation, bioreactor, vascular tissue engineering

## Abstract

Cardiovascular diseases remain a leading cause of mortality globally, with atherosclerosis representing a significant pathological means, often leading to myocardial infarction. Coronary artery bypass surgery, a common procedure used to treat coronary artery disease, presents challenges due to the limited autologous tissue availability or the shortcomings of synthetic grafts. Consequently, there is a growing interest in tissue engineering approaches to develop vascular substitutes. This review offers an updated picture of the state of the art in vascular tissue engineering, emphasising the design of scaffolds and dynamic culture conditions following a biomimetic approach. By emulating native vessel properties and, in particular, by mimicking the three-layer structure of the vascular wall, tissue-engineered grafts can improve long-term patency and clinical outcomes. Furthermore, ongoing research focuses on enhancing biomimicry through innovative scaffold materials, surface functionalisation strategies, and the use of bioreactors mimicking the physiological microenvironment. Through a multidisciplinary lens, this review provides insight into the latest advancements and future directions of vascular tissue engineering, with particular reference to employing biomimicry to create systems capable of reproducing the structure–function relationships present in the arterial wall. Despite the existence of a gap between benchtop innovation and clinical translation, it appears that the biomimetic technologies developed to date demonstrate promising results in preventing vascular occlusion due to blood clotting under laboratory conditions and in preclinical studies. Therefore, a multifaceted biomimetic approach could represent a winning strategy to ensure the translation of vascular tissue engineering into clinical practice.

## 1. Introduction

Cardiovascular diseases remain the leading cause of death in industrialised countries, accounting for an estimated 17.8 million deaths in 2017, with projections indicating a rise to 23.3 million annually by the year 2030 [1]. One of the most severe cardiovascular pathologies is atherosclerosis, a process that leads to the narrowing of arteries. In the case of coronary arteries, this leads to the weakening of the myocardium, i.e., the heart wall, eventually leading to myocardial infarction. As the disease progresses, there is a reduction in coronary blood flow and an alteration of fluid-dynamic conditions.

Once the narrowing of the arteries has progressed to the point where myocardial infarction is imminent or has already occurred, surgical intervention of aorto-coronary bypass becomes necessary [2]. Approximately 500,000 aorto-coronary bypass surgeries are performed annually in the United States. Bypass surgery is also performed to treat aneurysms or trauma. Currently, surgeons use either autologous tissue or synthetic biomaterials as vascular prostheses.

The transplantation of natural tissue, mainly the saphenous vein or internal mammary artery, is the preferred choice for coronary artery replacement. Indeed, the results of this procedure are quite favourable, with a success rate ranging from 50 to 70% [3]. Failures can be caused by various factors including intimal thickening (largely due to vessel adaptation in response to increased pressure and shear stress on the walls), vascular compression, inadequate vessel diameter, and disjunction near anastomoses [3]. However, many patients requiring aorto-coronary bypass surgery do not possess suitable autologous vessels, either due to illness or previous use during other procedures. Furthermore, even if available, the removal of autologous vessels from their native position in the vascular system is far from desirable. Given these considerations, it is clear how the need to construct alternative vascular substitutes for natural tissues arises.

The development of plastics and other polymers in the 1950s led to the use of the first synthetic materials to build vascular prostheses [4]. Such synthetic materials include polyesters, polyethylene terephthalate (PET, Dacron), and expanded polytetrafluoroethylene (ePTFE, Gore-Tex). However, the success rate of synthetic grafts was found to be substantially lower than that of natural vessels, particularly in small-diameter applications such as coronary arteries [5]. Issues associated with synthetic vessels include platelet adhesion, aggregation, and lower compliance compared to adjacent arterial tissues. The surface of synthetic materials is not as biocompatible as that of natural tissues, resulting in greater platelet activation and blood clotting. Despite their durability, synthetic materials can degrade over time, producing particles that may cause inflammation or other complications at the implant site. Unlike natural tissues, synthetic materials cannot integrate or reshape themselves within the body, potentially leading to long-term problems such as failure to adapt to changes in blood flow and pressure, and inability to grow with the patient, which is particularly relevant in pediatric cases. Additionally, synthetic materials cannot mimic the complexity of natural blood vessel functions, such as responses to vasodilation and vasoconstriction.

Conversely, allografts, although more biologically compatible than synthetic materials, can still trigger an immune response in the recipient, leading to transplant rejection. Even with immunosuppressive treatment, the risk of rejection cannot be completely eliminated. Allografts may lose their mechanical and functional properties over time, especially if not properly treated, and can undergo calcification, particularly if derived from animal tissues or inadequately processed. This calcification can result in loss of elasticity and vessel functionality. Although vessels treated with aldehydes to prevent reactivity have been used, they have exhibited thrombogenicity comparable to synthetic grafts. These challenges have driven the research and development of engineered vascular substitutes [6].

The fundamental concept of vascular tissue engineering involves designing a tubular scaffold using a biodegradable polymer which is then seeded with patient-specific cells. Following this, the graft matures within a dynamically simulated microenvironment before being implanted into the patient. Over time, the polymeric graft degrades with a precisely regulatable dynamic, allowing for the gradual replacement by a newly formed extracellular matrix (ECM). This process maintains the structural integrity of the graft while facilitating the development of native-like regenerated tissue. Compared with traditional prostheses, tissue-engineered vascular grafts (TEVGs) created in the laboratory can emulate the properties of autografts with remarkable accuracy.

Starting from the foundational work of Weinberg and Bell in 1986 [7], the field of vascular tissue engineering has demonstrated significant growth over the past few decades. Researchers have explored numerous approaches aimed at developing small-diameter blood vessels suitable for clinical applications, leading to substantial advancements in our comprehension of vascular biology and engineering principles.

Current research endeavours in this field are heavily focused on incorporating native-like features of autologous vessels into tissue-engineered grafts, to facilitate their clinical translation. As the development of biomimetic grafts requires a multifaceted approach, this review aims to provide an updated overview of the different biomimetic approaches investigated so far for vascular tissue engineering purposes, from scaffold material, architecture, and functionalisation cues, to bioreactor design. By these means, this review can significantly contribute to the existing literature by introducing novel insights and perspectives on multidisciplinary technologies that could facilitate vascular tissue engineering transition from bench to bedside.

## 2. Anatomy and Functions of Healthy Blood Vessels

The engineering of biomimetic blood vessel substitutes should begin with a careful understanding of the anatomy and functions of the native tissue.

Blood vessels constitute closed circulatory systems that penetrate most of the body tissues. They are categorised into arteries, capillaries, and veins, based on their structure and function [8]. Large vessels, such as arteries and veins, primarily facilitate efficient transport to distant sites, while small vessels like capillaries and arterioles enable optimal exchange of nutrients, oxygen, and waste within organs and tissues. Consequently, engineering large vessels (>6 mm) requires different design requirements and approaches when compared to manufacturing small vessels (<6 mm).

**Figure 1:**
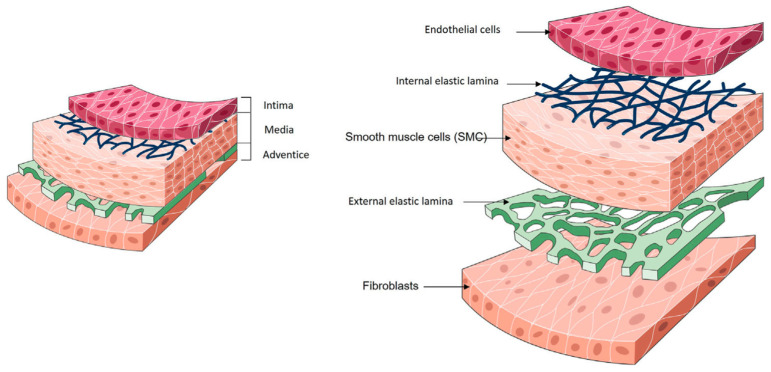
Anatomy of blood vessels. Reproduced from Devillard and Marquette [9], an open-access article distributed under the terms of the Creative Commons Attribution License (CC BY).

The wall of natural arteries comprises three distinct layers of tissue: the intima, the media, and the adventitia (Figure 1). Each layer contributes significantly to the vessel’s overall function, fulfilling essential roles in haemostasis, regulating vascular tone, and maintaining hemodynamic balance throughout the circulatory system. Collaboration between cells and ECM components is vital for preserving the dynamic environment of the artery wall.

The intima, the inner layer, is in direct contact with the blood. It is lined with a single layer of endothelial cells (ECs), collectively referred to as the endothelium. The primary functions of the endothelium include providing an anti-thrombogenic layer, preventing platelet aggregation, and regulating vascular permeability and homeostasis. In vivo, the endothelium is subjected to constant shearing forces caused by blood flow. In response to these forces, ECs elongate in the direction of blood flow. ECs produce their ECM, called the basal lamina, during development and in response to injury. This basal lamina acts as a continuous boundary between the endothelium and underlying structures. The intima is separated from the media by a thin layer consisting of elastin and type IV collagen (COL), known as the internal elastic lamina [10]. This lamina is porous, facilitating the diffusion of nutrients from the vessel lumen to the underlying tissues. The primary function of the internal elastic lamina is associated with maintaining the elastic resilience of the vascular wall to sustain blood pressure [11,12].

The media represents the middle layer. Smooth muscle cells (SMCs) constitute the predominant cellular population within the medial layer of blood vessels. Their main role is to synthesise ECM components and regulate vascular tone throughout the cardiac cycle. SMCs are influenced by the secretion of small molecules and growth factors by ECs, establishing a paracrine signalling loop between ECs and SMCs. The ECM composition of the medial layer consists primarily of COL and elastin. In the medial layer, SMCs, COL and elastin fibres are concentrically organised along the axis of the vessel, thereby representing the main component of the vessel’s mechanical strength [11,12].

The outer layer of the vessel is the adventitia, which is made of connective tissue and contains fibroblasts. Fibroblasts within the adventitia deposit an ECM that is rich in COL content, with collagenous fibres typically circumferentially arranged. Due to the high tensile strength of COL, its abundant presence, and its circumferential alignment, the adventitia emerges as the mechanically strongest layer in the vessel wall. Consequently, it plays a crucial role in preventing vessel rupture beyond physiological pressures. Additionally, the adventitia houses the vasa vasorum, a network of small blood vessels supplying nutrients and oxygen to all cells within the vessel wall [11,12]. In large-diameter arteries, an external elastic lamina is positioned between the media and the adventitia.

As it appears, vascular tissues represent intricate and constantly changing structures. In the vessel wall, the correct structure, composition, and function of each layer are vital for sustaining vascular homeostasis. The initiation and advancement of cardiovascular disease (CVD) disrupt the normal functioning of each layer, such as nutrient delivery and immune signalling, leading to severe clinical consequences. It is clear that the complexity of native blood vessels should be considered during vascular tissue engineering, as considering the recreation of all layers of the arterial wall, or at least the inner and middle layers, and their properties would be beneficial in obtaining a functional vascular substitute and achieving a good degree of success.

## 3. Challenges and Requirements of an Ideal Tissue-Engineered Blood Vessel

Although the engineering of blood vessels has significantly advanced since the first attempts, in the late 1980s, to produce the first in vitro TEVGs from vascular cells cultured on a COL matrix [7], several challenges still exist. Graft patency is still compromised due to thrombosis, most likely stemming from post-implantation endothelial cell retention or alteration of endothelial cell function following in vitro culture. Additionally, there is a possibility of graft failure due to post-implantation rupture in a physiological flow environment, which would lead to catastrophic consequences. Finally, it has been observed that the mechanical properties of engineered vascular grafts are inferior to those of natural arteries. Therefore, various approaches are being considered in terms of cell sourcing, scaffold materials, and culture conditions, to manufacture an optimal and clinically employable vascular substitute.

The ideal arterial substitute should fulfil the following criteria:

- Be biocompatible and non-immunogenic;
- Be resistant to infections and chronic inflammation;
- Offer an adequate microenvironment to support cell growth and ECM regeneration;
- Be able to acquire structural function immediately after implantation;
- Be devoid of cracks and thrombus-resistant, but with adequate porosity for effective metabolic exchange with the external environment, healing and angiogenesis. These attributes are generally provided by an artery possessing an intact endothelium, which also acts as a secretory tissue and barrier with selective permeability;
- Have appropriate mechanical properties (in terms of graft tensile strength, elastic modulus, burst pressure, compliance, and suture retention strength), in consideration of the high pressure to withstand after implantation [13]. Graft tensile strength is a crucial property in retaining graft integrity as it expresses the resistance of the graft to breakage caused by the mechanical forces, exerted on it during and after implantation. Elastic modulus is an equally critical parameter referring to the ability of the graft to mimic the elastic properties of natural blood vessels, which is essential for long-term functioning and compatibility with the cardiovascular system. The balance of these parameters is essential to guarantee adequate resistance and flexibility of the graft. Suture retention strength is also essential to retain sutures during the surgical procedure for implantation. Graft compliance was demonstrated to be beneficial for long-term patency, as compliance mismatch at the anastomosis between the native vessel and the engineered substitute is associated with adverse biological responses and long-term graft failure. A high burst pressure is fundamental to sustain physiologic variations in pressure without rupture. There is still a lack of consensus regarding its ideal value, as it depends also on the implantation site. In general, a burst pressure value above 2000 mmHg is desirable [14];
- Possess appropriate vasoactive physiological properties, including the ability to contract or relax in response to neural and chemical stimuli;
- Be economically manufacturable within a short timeframe, meeting various patient-specific parameters, such as diameter and length;
- Be able to remodel in vivo.

In native blood vessels, all of these ideal requirements are provided by the composition and arrangement of cells and ECM across the different layers of the blood vessel wall [15]. This is the reason why a biomimetic approach in blood vessel tissue engineering is paramount to obtaining a functional replacement.

## 4. Significance and Importance of Scaffold-Based Blood Vessel Tissue Engineering

A significant hurdle in vascular tissue engineering consists of identifying strategies for effectively distributing, arranging, and maturing different cell types within a tubular structure. One of the most significant challenges in creating blood vessels lies in effectively distributing cells within vascular tissue to mimic their natural arrangements [16]. Various techniques have been devised to tackle this challenge, for instance by employing cell sheet engineering techniques, or by exploiting the predefined ECM organisation by decellularising vascular tissue sourced from xenogeneic origins and using them as support for subsequent cell culture. Another investigated strategy is the creation of scaffolds using methods such as electrospinning or moulding, followed by cell seeding [9]. Scaffold-based tissue engineering strategies involve utilising a scaffold material to provide a structural framework for cell attachment, proliferation, and differentiation. Scaffolds can be fabricated using both natural and synthetic polymers. An important advantage of scaffold-based approaches is their ability to guide cell colonisation and proliferation along predefined pathways within the scaffold structure. This can help mimic the natural organisation of vascular tissues and promote the formation of functional blood vessels. [17]. Scaffold-based methods are therefore receiving significant attention due to their versatility, the wide availability of both synthetic and natural polymers, and the obtainable three-dimensional porous structures, which facilitate cell growth, nutrient diffusion, tissue regeneration, and scaffold biodegradation [18,19,20,21,22,23,24,25].

The report by Niklason et al. [26] marked a significant milestone in scaffold-based vessel tissue engineering, particularly in the field of small-diameter blood vessel regeneration. By seeding bovine aortic SMCs onto a polyglycolic acid (PGA) scaffold and culturing them dynamically in a bioreactor for 8 weeks, they were able to obtain an engineered tissue with mechanical properties comparable to native vessels. This interesting result has stimulated research on scaffold-guided vessel regeneration, especially for small-calibre vessels. However, to date, there are no clinically available scaffolds for tissue engineering of small-diameter blood vessels.

Certainly, significant progress has been made by moving on from monolayer scaffolds to multilayer scaffolds, given that the arterial wall is an intrinsically multilayered structure in which each layer performs specific functions. In recent years, there has been a shift towards accurately mimicking the native arterial geometry, as will be further discussed in Section 6. This approach aims to address the limitations of current scaffold-based strategies by better replicating the complex structure and functionality of natural blood vessels. By developing multi-layer scaffolds that emulate the distinct properties of each layer in the arterial wall, scientists hope to improve the outcomes of tissue engineering for small-diameter blood vessels [27].

### 4.1. Scaffold Cell Seeding

Once the scaffold for vessel regeneration has been produced, the next particularly critical step is correct cell seeding, to produce a system capable of reproducing vascular functionality.

Static seeding, a standard procedure, comes with drawbacks, including the uneven distribution of cells within the scaffold, a strong reliance on cell migration within the scaffold, and the porosity of the biomaterial utilised to construct the scaffold [28]. To address the shortcomings of static seeding, dynamic seeding techniques have been devised, notably through the implementation of rotational systems [29] in which centrifugal forces aid in the uniform transfer of cells into a porous scaffold. Researchers have observed improved seeding efficiency and homogeneity compared to static or spinner flask techniques. However, they also acknowledged a potential negative impact on cellular viability and morphology, although the extent of this impact was not quantified.

An alternative seeding method has been devised by utilising magnetic fields. This approach relies on directing magnetically labelled cells through a magnetic field gradient, facilitating their seeding into tubular structures [30]. The different vascular layers are created by repeating this procedure with various cell types.

As an alternative to in vitro cell seeding, in vivo scaffold cellularisation and tissue growth were investigated. In this approach, a scaffold without cells is implanted into the host, where it should promote cell colonisation [22,31,32,33,34,35,36]. Unfortunately, the major limitation of this method is that there is a lack of control over the interaction of the scaffold with the different types of host cells, therefore it is not possible to predict what may happen to the scaffold and processes like fibrosis, thrombosis, pore obstruction and slow degradation can occur [37,38,39].

### 4.2. Moulding Cellularised Biomaterials

Several studies have demonstrated that moulding cellularised biomaterials can enable the manufacturing of custom-shaped structures [40,41,42,43]. This technique involves pouring a biomaterial solution into a mould with a custom external shape, allowing it to solidify or crosslink, and then removing the mould to obtain the desired structure. The versatility of this method lies in its ability to produce a wide range of shapes, simply by modifying the mould architecture.

A step-by-step casting technique was applied by Helms et al. to create a tri-layered blood vessel [44]. They first differentiated adipose-derived stem cells (ASCs) into SMCs, which were then embedded into a tubular fibrin matrix compacted in a mould to form the media layer. Then, a fibrin matrix was moulded around the media layer to mimic the adventitial layer. Finally, human umbilical vein endothelial cells (HUVECs) were seeded onto the luminal surface to cellularise the intimal layer. However, because of the static nature of the process, a non-uniform seeding of the cells was obtained, leading to an uneven cell distribution within the structure.

Another moulding technique was developed by Keita Kinoshita et al., who fabricated multilayered vascular tissue models by depositing cell layers in an agarose hydrogel mould [41]. SMCs were embedded into an agarose mould, and ECs were seeded on the lumen. This method allowed for the creation of complex-shaped constructs, but the problem of cell distribution inhomogeneity persisted.

Therefore, at the moment, it can be said that while moulding cellularised biomaterials allows for the fabrication of customised structures, challenges such as non-uniform cell distribution and gelation homogeneity need to be addressed to optimise the technique for tissue engineering applications.

### 4.3. 3D Bioprinting

The technique known as 3D bioprinting enables the fabrication of intricate and functional heterocellular structures, offering the potential for precise deposition of cells with anatomical morphology [45]. The bioink used in 3D bioprinting is represented by cell-laden hydrogels, where hydrogels serve as a scaffold to support cells. More recently, aggregates of thousands of individual cells called spheroids have emerged as a bioink for application in 3D bioprinting. Spheroids are deemed an optimal bioink since they enable cells to establish cell–cell junctions and construct their ECM [46].

In 2009, a study was conducted [47] in which an attempt was made to bioprint small-calibre vessels using spheroids of human skin fibroblasts bioprinted on agarose-based rods. The same researchers also demonstrated the construction of a vascular tube consisting of an internal layer generated from human umbilical vein SMC spheroids and an external layer obtained from human skin fibroblast spheroids.

Another research group created multicellular spheroids in 2015 [48] containing HUVECs (40%), human aortic SMCs (10%), and human dermal fibroblasts (50%). These multicellular spheroids were inserted into a needle array where they formed tubular structures. After four days of incubation, the needle array was removed and the tubular structures were perfused within a bioreactor for another four days. These TEVGs were successfully implanted into the abdominal aorta of nude rats and remained patent until the study endpoint, postoperative day 5. Histological examination revealed post-implantation endothelialisation. Recently, the same research group introduced a method to cryopreserve spheroids using a 10% Me_2_SO_4_ solution that would enable large-scale production of spheroids, increasing the potential feasibility of 3D bioprinted TEVGs for clinical use [49].

Today, various bioprinting techniques are available; however, not all of them are suitable for producing vascular tissues, comprising three different cell layers within tubular architectures. The most suitable technology is microextrusion-based bioprinting [50], a method in which a bioink containing an appropriate biomaterial and cells is loaded into a printing cartridge and extruded through a nozzle using a mechanical or pneumatic system. Microextrusion-based bioprinting offers the advantage of utilising a wide range of biomaterials and cells, which aids in replicating the complexity of vascular tissue structure.

Another technique facilitating the rapid and straightforward reconstruction of a multi-layered vascular tissue is the coaxial technique [51,52,53], which employs specialised coaxial nozzles. This method enables the encapsulation of one bioink within another and it is particularly useful in vascular tissue engineering for creating multiple concentric layers within a tubular structure.

A triple coaxial bioprinting technique was used to construct a biomimetic blood vessel using vascular tissue-derived extracellular matrix (VdECM) mixed with vascular smooth muscle cells (VSMCs) and alginate mixed with ECs as bioinks, to form the media and intima layers, respectively. After 3 days of static culture and 2 weeks of maturation under pulsatile stimulation, the constructs, cross-linked with a CaCl_2_ solution, were fixed for histological analysis and good endothelialisation was demonstrated. The authors also evaluated the constructs in vivo using a rat model and observed that the implants remained patent for 3 weeks [52]. The triple coaxial bioprinting technique was further implemented by the same group, printing directly into a pre-gel bath containing fibroblasts [54]. In this way, the adventitia layer (which was missing in the previous system) was formed around the extruded tube.

While the coaxial extrusion technique currently stands as the most advanced method for generating vascular tissue with a three-layer structure, limitations persist concerning the dimensions and architecture of the produced vessels. These constraints primarily stem from the size of the coaxial nozzle.

## 5. Biomimicry in Scaffold Material

In recent years, research activity related to the design of scaffolds has moved in the direction of replicating the native characteristics of arteries. The development of a scaffold capable of mimicking the structure–function relationships present in the arterial wall offers significant advantages, particularly in addressing key limitations observed in current synthetic grafts, such as mechanical incompatibilities and thrombogenicity [27]. The biomimetic approach could be particularly advantageous in the engineering of small-diameter functional blood vessels. Although biomimicry is not a new concept, it has garnered considerable attention in tissue engineering in recent years. To date, synthetic and/or natural materials have been used in the literature to create double- and trilayer scaffolds [54]. However, to create a structure that closely mimics natural tissue, the use of natural materials (such as natural polymers and decellularised matrix) proves advantageous. Various multilayer approaches, which exclusively employ natural materials, and hybrid approaches that incorporate both synthetic and natural materials have been reported in the literature [27]. An overview will be provided in the next paragraphs.

### 5.1. Natural Polymers

Natural polymers, in particular ECM proteins, such as COL, fibrin, elastin, etc., have been widely investigated for the production of blood vessel scaffolds, thanks to their biocompatibility and numerous bioactive cues. These proteins offer a favourable 3D microenvironment with suitable binding sites for cellular populations, and various methods can be used for the proper production of tubular scaffolds by employing these proteins, such as electrospinning, freeze-drying, and mould casting [55,56,57,58,59,60,61,62,63,64,65].

Among these ECM proteins, COL has been used for decades to produce TEVGs [42,55,56,66,67]. COLs are a family of particularly abundant proteins that can be easily isolated, manipulated, and used for the production of scaffolds. COL I is the most abundant type in mammalians and offers a great number of integrin-binding sites, which can control cell adhesion, differentiation, and overall cellular behaviour. Several studies have attempted to replicate the features of blood vessels through the production of scaffolds using COL, but the results were less than satisfactory due to the low mechanical properties of COL gels [55,66]. The mechanical properties of COL-based scaffolds can be improved by cross-linking. However, the use of chemical cross-linking agents, such as glutaraldehyde (GTA), while improving the mechanical properties, leads to severe cytotoxicity risks [55,66].

Alternative strategies to chemical cross-linking have been developed to enhance the poor mechanical properties of COL-based TEVGs, resulting in notable improvements in the integrity of the grafts [66,67,68,69]. Alternative methods of chemical cross-linking include physical and enzymatic approaches. UV or Gamma radiation can be used to induce cross-linking through the formation of free radicals that create covalent bonds between COL molecules. The thermal dehydration-based method involves heating COL under moisture-free conditions to promote cross-linking of the fibres. Freeze-drying followed by heating can also lead to the physical cross-linking of COL fibres, improving the mechanical stability of the material. Enzymatic methods instead involve the use of enzymes, such as transglutaminase, which catalyses the formation of covalent bonds between the amino groups and the carboxyl groups of COL molecules, creating a stable and biocompatible network, or such as Lysyl oxidase, which can be used to induce cross-linking, exploiting the natural stabilisation mechanisms of COL [66,67,68,69].

Strategies incorporating cells, matrix components, and intracellular biomolecules have also been employed to enhance the mechanical strength of COL-based constructs through the compacting and reorganisation of COL fibril architecture. Seliktar et al. [56] have shown that seeded cells and mechanical conditioning can rearrange COL fibrils circumferentially, resulting in increased strength. However, currently, the obtainable tensile strengths, burst pressures, and strength at the anastomosis of COL grafts remain much lower than those of native vessels.

Another natural polymer that has emerged as an interesting alternative to COL is fibrin, a protein produced through the cleavage of fibrinogen [70] and a rich source of growth factors, cytokines, and chemokines. Its natural role in wound healing, widespread clinical acceptance as a tissue sealant, and its potential to generate an autologous biomaterial from the patient’s blood make it particularly appealing [71].

The first employments of fibrin involved the creation of coatings in COL-based vascular grafts [72]; subsequently, considerable efforts were made to develop vascular grafts based solely on fibrin [60,62]. It has been observed that these grafts require a pulsatile bioreactor system for correct maturation. Approaches involving the use of ECs and SMCs in fibrin-based vascular grafts have also been proposed [73]. Swartz et al. [61] analysed recellularised fibrin-based vascular grafts exhibiting compromised biomechanical properties, which possessed an average burst pressure of 543 ± 77 mmHg, insufficient to withstand the physiological pressures of blood flow. A recent study by Yang et al. [74] demonstrated that the mechanical properties of these grafts can be improved by incorporating polycaprolactone (PCL). Electrospun hybrid PCL/fibrin vascular grafts were tested for mechanical properties, cytotoxic effects, and biocompatibility in vivo. The burst pressure of these hybrid vascular grafts was 1811 ± 101 mmHg, similar to native blood vessels (2000 mmHg), and no cytotoxic effects or immune responses were reported in vivo. Furthermore, fibrin gels have been shown to stimulate SMCs to synthesise elastin, a vital component of the artery wall often overlooked in many COL-based tissue-engineered blood vessels [55].

Elastin is another interesting protein for vascular scaffold fabrication purposes, considering its role in native vessels. Elastin plays a crucial role in preserving the elasticity of blood vessels, preventing dynamic tissue creep by stretching under pressure and reverting to its original shape after the load is removed [75]. This property helps prevent permanent deformation under pulsatile flow and is advantageous for maintaining long-term compliance. Additionally, elastin exhibits anti-thrombogenic and anti-inflammatory properties, further contributing to the functionality of blood vessels. These properties are particularly essential for small-diameter TEVGs [63]. Similar to COL, elastin can enhance EC proliferation, viability, and endothelialisation of the lumen surface [76]. Besides providing cell-binding sites, elastin is also associated with activating pathways that regulate the proliferation and differentiation of vascular cells. Specifically, elastin has been shown to stimulate the gene expression of SMC markers in vitro [77,78] and it was found to modulate the phenotype of SMCs towards a contractile state [79].

The long-term failure of TEVGs is primarily linked to thrombosis, intimal hyperplasia, and aneurysm formation, which are often attributed to the scarcity of elastin.

To address the issue of constructive remodelling of implanted TEVGs, current approaches focus on incorporating elastin into the grafts before implantation. Therefore, one potential strategy is to cultivate SMCs in TEVGs to enable the synthesis of new elastin within the grafts. However, adult SMCs have limited capacity for elastin production, prompting researchers to turn to stem cell-derived SMCs which show promising improvements in tropoelastin production and assembly in response to biomechanical cues [80]. In an ideal scenario, a biodegradable TEVG would be seeded with patient-specific stem cells-derived SMCs and matured in a dynamic bioreactor environment alongside bioactive factors (transforming growth factor-beta 1 (TGF-β1), fibronectin, NO, miRNA-29A, and stromal vascular fraction (SVF) [65]), which stimulate the production of elastin by SMCs. This approach could produce an implantable graft with sufficient and well-organised elastin, thereby enhancing its functionality and durability in vivo. Alternative solutions involve integrating engineered elastin, such as recombinant tropoelastin and elastin-like recombinamers, into TEVGs. Another approach is to incorporate insoluble elastin extracted from native arteries into TEVGs. Utilising native decellularised vessels that maintain elastin organisation is another viable option. These strategies aim to enhance the elastin content and organisation within TEVGs, ultimately improving their performance and biocompatibility in vivo.

Natural polymers which are not derived from the ECM, such as chitosan (Ch) and silk fibroin (SF), have also found extensive use in the production of TEVGs. Systems based on Ch and SF are characterised by easily controllable mechanical properties. Ch is a linear polysaccharide derived from the shells of shrimps and crabs and finds widespread application in tissue engineering. It has mild antibacterial properties, making it advantageous for in vivo use. In scaffold fabrication, Ch can be blended with degradable polymers like PCL and polylactic acid (PLA). In vascular graft development, electrospinning technology is used to create conduits with favourable properties for cell adhesion and proliferation. In a recent study [59], it was observed that by regulating the concentration of Ch it was possible to obtain a balance between adequate mechanical resistance to physiological stress and adequate compliance to prevent adverse hemodynamic conditions. Wang et al. realised a PCL/Ch hybrid vascular graft with anti-thrombogenic and antibacterial properties. Similarly, Yao et al. developed electrospun PCL/Ch grafts combined with heparin (Hep-PCL/Ch), enhancing patency and endothelialisation in vivo. These findings suggest the potential of Ch in crafting functional small-diameter vascular grafts (SDVGs) when combined with degradable polymers.

SF is also attractive because it is compatible with various manufacturing processes, including electrospinning, spinning and weaving techniques. Its thickness can vary greatly and it has customisable mechanical properties, a slow degradation rate, and good biocompatibility for cell infiltration. Fibroin has antithrombogenic properties and can degrade over time. Enomoto et al. [81] successfully developed fibroin-based SDVGs and compared their patency with ePTFE vascular conduits in vivo. They showed that, for over 64 weeks, fibroin-based SDVGs remained patent at a rate of 85%, while ePTFE grafts remained patent only 48% of the time. Furthermore, a greater number of SMCs and ECs were observed in fibroin-based SDVGs compared to ePTFE grafts, indicating better overall functionality of the former.

Blends of natural polymers, of both protein and polysaccharide type, can also be used to produce a scaffold more closely mimicking the composition of the natural vascular ECM, as proposed by Rosellini et al. [82]. Tubular scaffolds were produced using a specially designed mould, starting with a gelatin/gellan/elastin blend, selected to mimic the composition of the ECM of native blood vessels. Scaffolds were obtained through freeze-drying and subsequent cross-linking. Results of the in vitro characterisation showed good porosity, which could promote cell infiltration and proliferation, and a dense external surface, which could avoid bleeding. Moreover, the developed scaffolds showed good hydrophilicity, an elastic behaviour similar to natural vessels, suitability for sterilisation by an ISO-accepted treatment, and adequate suture retention strength. Additionally, the inclusion in the blend of a polysaccharide component increased the number of functional groups available for subsequent scaffold functionalisation.

### 5.2. Decellularised Tissues

Decellularisation is among the most used methods to produce vascular scaffolds. It consists of removing the totality of the cells from the tissue while maintaining its ECM intact by preserving its shape and characteristics [83]. The preservation of the natural components of vascular ECM confers a natural structure and native vessel-like mechanical properties to the resulting scaffold. Furthermore, proteins and glycosaminoglycans (GAGs) located in the natural vascular ECM provide various cell adhesion and development domains [73]. Decellularised scaffolds can be obtained from various sources, both allogeneic and xenogeneic, such as animals (e.g., porcine, sheep) or cadaver donors when effectively decellularised and used immediately. However, the use of animal-derived grafts poses challenges due to the potential immune response triggered by the presence of alpha-gal epitopes (Galalpha1-3Galbeta1-(3)4GlcNAc-R) in non-primate tissues. Efforts to mitigate this immune response include cleaving alpha-gal epitopes or using transgenic animals lacking these epitopes [84]. Cellular and antigenic material must be completely removed to avoid the risk of the immune and/or inflammatory response [27,85]. Vascular grafts have been obtained starting from various types of decellularised vessels such as carotid artery [86], aorta [87,88], internal mammary artery [89], umbilical artery [90], saphenous vein [91], coronary artery [92], femoral vein [83], and vena cava [93]. Human-derived vascular grafts, obtained from cadaver femoral veins or umbilical vessels, have also been explored. While human umbilical arteries (hUAs) offer potential as SDVGs, challenges such as technical difficulties and lack of elasticity need to be addressed. Nonetheless, efforts to produce decellularised hUAs have shown promise, with studies demonstrating long-term patency and successful in vivo remodelling [94,95,96].

The protocols for vessel decellularisation include the use of chemical methods, physical methods, and enzymatic methods. For instance, surfactant detergents such as sodium dodecyl sulphate (SDS), TritonX-100 (TX) and tributylphosphate can be used, but also different types of enzymes such as DNase and trypsin. Detergents serve to remove cells from tissues through the solubilisation of the lipid bilayer of cell membranes and the dissociation of DNA from proteins [97]. On the other hand, enzymes are responsible for the proteolysis process. Trypsin acts on native COL and elastin, while nucleases, such as DNase, cleave nucleic acid sequences and can assist in the removal of nucleotides after cell lysis [97]. In addition to these agents, hypotonic and hypertonic solutions can also be utilised. Hypotonic solutions induce cell lysis through simple osmotic effects, while hypertonic saline solutions dissociate DNA from proteins [97]. For cell lysis, a freeze–thawing method can also be used, given its effectiveness in preserving the nature of ECM components (e.g., COL, elastin, and fibronectin) and the native mechanical strength when compared to detergent-based methods [98]. Nonetheless, it should be followed by further treatment since it does not allow the complete removal of all cellular components (e.g., 90% of DNA) [99]. The success of a protocol for vascular decellularisation stems from the use of adequate quantities of agents and treatment repetitions to allow the complete removal of cells without damaging or altering the ECM. The choice of the best method depends on various factors, such as original tissue thickness, cellularity, density and lipid content. Ineffective decellularisation may lead to inflammatory response and antigenicity, increasing the risk of graft failure [87]. Therefore, it is vital to adhere to specific criteria to ensure satisfactory tissue decellularisation. According to Crapo et al., decellularised tissues, including vessels, should exhibit < 50 ng dsDNA/mg ECM dry weight, <200 bp DNA fragment length, and a lack of visible nuclear material in stained tissue sections [97].

Some investigations employ physical methods alongside chemical or biological agents to enhance the penetration and efficacy of decellularisation in vascular tissue [100]. For instance, Simsa et al. examined the decellularisation of vena cava using TX-100, tri(n-butyl) phosphate (TnBP), and DNase under static, agitation, and perfusion conditions. They found that all three methods effectively reduced DNA content, with agitation and perfusion at velocities up to 100 mL/min proving optimal for promoting vessel decellularisation [93]. In their investigation, Omid et al. also combined chemical and physical decellularisation techniques on ovine coronary arteries. By inducing osmotic pressure with hypertonic/hypotonic solutions, cellular membranes were dissociated and removed effectively without the need for detergents or other chemical agents. A solution of 0.025% trypsin was then used to eliminate any remaining debris, followed by a final cleansing with 1% TX-100. Performing all decellularisation steps at 4 °C resulted in minimal damage to the ECM ultrastructure, as evidenced by similar mechanical strength and swelling ratio to native vessels, as well as robust cell attachment, migration, and proliferation observed via optical and scanning electron microscopy (SEM). Furthermore, cytotoxicity assessments via MTT assay confirmed the absence of cytotoxic effects, and the resulting biological scaffold proved to be storable at −20 °C. This decellularisation method yielded a biological scaffold with intact ultrastructure which meets both mechanical and cellular property requirements [101].

Evaluation of decellularised vascular scaffolds should also extend beyond cell removal and mechanical properties to include cytotoxicity and cytocompatibility assessment. For example, Campbell et al. decellularised porcine coronary arteries and seeded them with bovine aortic ECs and SMCs, resulting in successful cell adherence and endothelial layer formation [102]. Similarly, Lin et al. observed the adhesion and proliferation of rat ASCs and HUVECs on decellularised arteries and demonstrated in vivo endothelisation in a rat abdominal aorta repair model [92].

Lastly, to enhance scaffold success, researchers have modified decellularised vessel surfaces with biomolecules like heparin (Hep) to improve endothelisation and prevent hyperplasia and thrombogenesis [85,88,103]. Dimitrievska et al. immobilised Hep on decellularised aorta arteries, reducing platelet adhesion and supporting HUVEC adhesion and proliferation [103].

Refinements in the decellularisation process have led to the near-complete removal of immunogenic components; however, achieving physiologically relevant mechanical properties without further processing, such as maturation through a bioreactor, remains a challenge [27]. Despite progress in decellularisation, several factors contribute to this difficulty. Firstly, there is no universal standardised protocol for decellularisation, and different methods can variably affect the structure and mechanical properties of the tissue. Additionally, cell repopulation and remodelling are complex processes that do not always succeed in fully restoring the physiological mechanical properties. Lastly, the intrinsic variability between tissue samples, due to genetic differences, age, and health status of donors, can influence the mechanical properties of decellularised tissues. Nonetheless, interesting approaches have been adopted. For instance, Gong et al. [104] adopted a unique approach by decellularising a rat aorta to preserve its histocompatibility and biomechanical properties, followed by electrospinning a circumferentially aligned layer of PCL to reinforce the decellularised vessel. The donor aortic vessels underwent decellularisation using a combination of detergents and were dehydrated through vacuum freeze-drying. PCL nanofibres were electrospun around the acellular aortic grafts to reinforce the decellularised matrix. Mechanical testing demonstrated that the addition of electrospun PCL significantly enhanced the biomechanics of the decellularised vessels. Vascular ultrasound and micro-CT angiography confirmed satisfactory patency of all grafts implanted in a rat model for up to 6 weeks. Furthermore, electrospun PCL effectively prevented vasodilation and aneurysm formation post-transplantation and reduced inflammatory cell infiltration. Building upon this work, other groups have reinforced decellularised vessels with electrospun layers, offering promising results by combining the biological advantages of natural materials with improved mechanical properties [104].

Another strategy to enhance the mechanical properties and patency of decellularised vessels involves re-cellularisation. A complete, continuous, and functional layer of ECs along the vessel wall is essential for the safe use of decellularised scaffolds in clinical applications. Thus, identifying the best cell source and the optimal recellularisation process is of great importance. The choice between in vitro pre-seeding and in vivo cell proliferation for decellularised engineered vascular grafts depends on various factors, including the efficacy of regeneration, the immune response, and the complexity of the process. In vitro pre-seeding offers several advantages: precise control of culture conditions, uniform distribution of cells, real-time monitoring and optimisation, and the ability to evaluate tissue functionality before implantation. However, it is a complex procedure involving a greater risk of contamination and longer preparation times. Conversely, in vivo cell proliferation is simpler and less expensive, with reduced risks of contamination and a natural regeneration response. Nevertheless, it offers less control over environmental conditions, potentially adverse immune responses, and longer, less predictable regeneration times [83,91]. In vivo performance studies have shown that, while decellularised vascular grafts lack proper function and are prone to thrombus formation and graft failure, cellularised engineered grafts exhibit improved properties, including a lower risk of thrombus formation and rejection. Recellularised TEVGs with uniform coverage of ECs and VSMCs offer superior outcomes compared to non-cellularised grafts [27,83,105]. Various cell types, including peripheral blood mononuclear cells, ECs, and endothelial progenitor cells (EPCs), have been used for reseeding to promote patency. In a particularly promising study, pre-seeding resulted in 100% patency after 30 days of implantation [105]. However, long-term patency studies exceeding one year are lacking.

Despite advances in decellularisation, mechanical reinforcement strategies, and cell reseeding, the clinical use of decellularised vessels remains limited by several factors. Batch-to-batch variability in mechanical, biochemical, and biological properties—influenced by donor age, health status, and manufacturing processes—poses a significant challenge [104,105,106,107]. One issue with using decellularised vessels is the potential for limited recellularisation in living organisms, possibly due to the vessel wall’s dense ECM or chemical damage to the ECM during the decellularisation process. The limited success of currently available commercial decellularised grafts can be partly attributed to their lack of cellularity upon implantation. A functional endothelium, which is is essential to prevent thrombosis in smaller calibre vessel grafts, is particularly important for decellularised grafts with their exposed COL luminal surface [83]. While studies have shown promising patency rates for implanted decellularised vessels, they have not demonstrated superiority over synthetic grafts [5,6,104,108]. Thus, further research is needed to address these limitations and optimise the clinical utility of decellularised vessels.

## 6. Biomimicry in Scaffold Architecture

An ideal TEVG is biocompatible, matches the mechanical properties of native vessels, and promotes endothelialisation and tissue integration. Mimicking the structure of native arteries, with layers such as tunica intima, tunica media, and adventitia, is crucial for functional replication. Various techniques, including electrospinning, self-assembly, and 3D bioprinting, have been explored for TEVG fabrication. However, the challenge remains in replicating the intricate structure of the native ECM, which is pivotal for functional tissue regeneration [100,109,110].

Electrospinning offers a method to fabricate fibrous scaffolds with controlled architecture and mechanical properties resembling the native ECM. These scaffolds provide a high surface-to-volume ratio and adjustable porosity, promoting cell adhesion and angiogenesis [111]. Multilayered scaffold designs aim to mimic the hierarchical organisation of blood vessel walls and incorporate various materials or functionalities to control cell behaviour and tissue development [27,110]. Additive manufacturing technologies such as 3D printing allow precise control over the geometry of the scaffold and the distribution of bioactive components, facilitating cellular organisation, vascular network formation, and tissue maturation [110]. Furthermore, the design of TEVGs must consider the microstructure of native arteries, incorporating suitable porosity and pore sizes to facilitate cell migration and metabolic exchange. Bioprinting technologies, including extrusion and light-based approaches, offer the possibility to fabricate complex, patient-specific tubular structures with high precision and reproducibility, enhancing the translatability of TEVGs to clinical settings [27,100,110].

In summary, the quest for small-calibre vascular grafts has spurred innovative methodologies in tissue engineering, driven by the need for alternatives to autologous grafts with improved patency rates and reduced complications. Mimicking the complex structure and function of native vessels remains a key challenge in TEVG development, emphasising the importance of biomimetic approaches and advanced scaffold design techniques.

### 6.1. Fibre-Based Scaffolds

Fibre-based scaffolds fabricated through electrospinning techniques have garnered significant attention in the field of vascular tissue engineering due to their ability to mimic the intricate structure of native ECM. It is important to evaluate the technical intricacies of controlling topological fibre arrangement in electrospun tubular scaffolds tailored for vascular applications. Processing parameters such as applied voltage, feed rate, needle-to-collector distance, and mandrel rotation speed affect the final properties of the scaffold [100]. In addition, it is important to consider not only the technical aspects of scaffold fabrication but also the functional requirements for the production of effective tissue-related constructs. The microstructural requirements, including porosity, pore size, and interconnectivity, are crucial in facilitating cell movement and metabolic sharing, which are critical for tissue integration. Electrospinning emerges as a promising technique for precisely controlling the microstructure of scaffolds, particularly by regulating pore size and fibre alignment, which are critical for promoting tissue healing and integration after implantation. Electrospinning provides precise control over fibre diameters (50–500 nm) and mimics the ECM architecture of natural blood vessels. Challenges include poor cell penetration, surface properties that affect viability, and difficulties in regulating mechanical properties [100,109].

**Figure 2:**
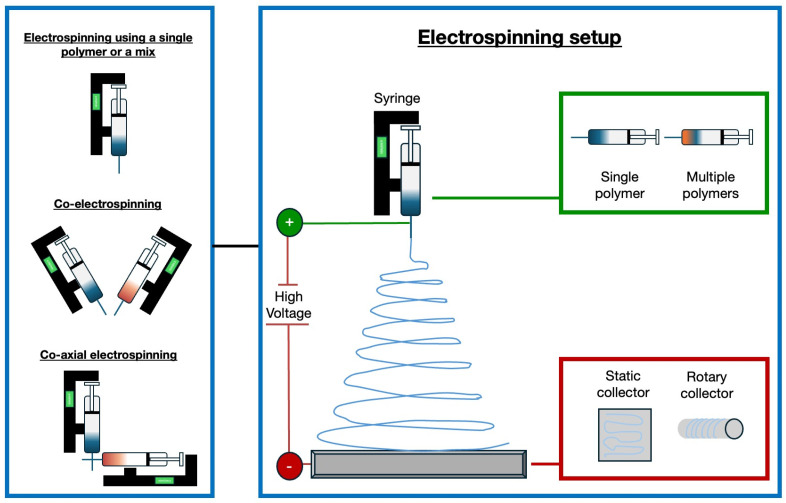
Experimental set-up for the production of tissue-engineered blood vessels using electrospinning. Various approaches are employed: a single polymer or a blend of polymers can be dispensed from a single syringe, or two distinct polymer filaments can be electrospun simultaneously using the co-electrospinning technique [113]. Coaxial needles are utilised to enable the electrospinning of two different materials via the co-axial electrospinning method, resulting in the formation of core-shell filaments. Different types of collectors can be used for the deposition of fibres, including static and rotating collectors [114].

In recent years, significant progress has been made with electrospun vascular grafts (ESVGs). They have evolved from single-layer, single-component structures to multi-layer, multi-component designs. These grafts can be produced by spinning fibres onto a rotating mandrel or by forming a sheet that is subsequently rolled up. The production of tissue-engineered blood vessels by electrospinning may involve the use of a single polymer or a polymer mix, the use of two different polymers by a co-electrospinning process, or the use of two materials electrospun through coaxial systems [112]. Figure 2 illustrates the electrospinning technique and possible variations in the setup to fabricate tissue-engineered blood vessels.

The most investigated materials to produce vascular scaffolds by electrospinning include gelatin (Gt) [115] and PCL, among synthetic components [116].

For example, Kong et al. [113] developed biomimetic Gt/PCL composite nanofibres containing different amounts of chondroitin sulfate (CS) via electrospinning technology. All solutions were electrospun at room temperature and controlled humidity. The resulting nanofibres exhibited suitable porosity (~80%) and could absorb PBS solution up to 650%. Composite nanofibres of Gt/PCL with a specific proportion of CS demonstrated reduced thrombogenicity and enhanced EC response, suggesting their potential as a scaffold for tissue engineering in blood vessel repair and regeneration.

Elsayed et al. [114] developed a new approach for creating electrospun Gt fibre scaffolds, with a precisely controlled fibre orientation and optimised Gt cross-linking, to achieve not only compliance equivalence with the coronary artery but also resistance equivalence of the wet tubular acellular scaffold (swollen with absorbed water) to that of the tunica media of coronary artery in both circumferential and axial directions. Solutions containing Gt type A in 2,2,2-Trifluoroethanol (TFE) were electrospun. To create scaffolds with multi-layer fibre alignment at ±45 degrees, spinning occurred for 4 min before pausing to flip the collector by 180 degrees. Most importantly, this study represents the first case of high suture retention strength among natural scaffolds and, in particular, among Gt scaffolds. Suture retention strength in the range of 1.8–1.94 N was achieved for wet acellular scaffolds, which is equal to or better than that of a saphenous vein. The produced scaffolds showed exceptional efficacy in promoting human SMC proliferation. SMCs seeded onto the upper surface adhered, elongated, and aligned with nearby fibres, aiding their migration throughout the scaffold thickness.

Another interesting study in which, through the electrospinning technique, nanofibrous tubular scaffolds were obtained was presented by Zhu et al. [117]. They proposed a polyurethane/Gt nanofibrous tubular scaffold, obtained through electrospinning and Hep grafting, which demonstrated improved hydrophilicity, adequate mechanical properties, and enhanced blood compatibility. In vivo studies confirmed that the obtained nanofibrous tubular scaffolds effectively promoted vessel regeneration, closely mimicking natural vessels, thereby holding significant potential for the developed biodegradable composite vascular grafts to prevent intimal hyperplasia and acute thrombosis.

Various studies in the field of tissue engineering depict electrospinning as a powerful technique to produce tailored scaffolds for the tissue engineering of blood vessels, offering precise control over structure and alignment and enhancing their efficacy in promoting vascular regeneration. In particular, thanks to its versatility, electrospinning has also been largely investigated to produce two-layer and three-layer scaffolds, mimicking the multilayer architecture of native vessels, which will be further discussed in the next two sections.

### 6.2. Two-Layer Scaffolds

While various techniques, including cell sheets and synthetic or natural biomaterial platforms, have been explored, scaffold-based methods stand out due to their versatility and the abundance of usable synthetic and natural polymers. Biomimicry, and particularly structural biomimicry, plays a significant role in this domain, aiming to replicate the structure–function relationships of natural tissues. The multi-layered scaffold approach represents a solution to mitigate the primary shortcomings of existing synthetic grafts, namely, their mechanical incompatibility and propensity for thrombosis [100].

The first attempts in this direction are represented by the development of bi-layer scaffolds, obtained from both synthetic and natural materials [6,27,109,110,111,112].

While scaffolds made entirely of natural materials are rare due to their lack of mechanical strength, silk stands out as an exception. Liu et al. [118] demonstrated the potential of silk-based scaffolds, utilising heparinised braided silk tubes as a non-thrombogenic solid inner layer, complemented by an SF microporous sponge cast through lyophilisation as the outer layer. Compared with previously fabricated silk-based vascular scaffolds, the fibre reinforcement provided a comparable or higher mechanical strength, burst pressure, and suture retention strength, as well as mechanical compliance, to saphenous veins for vascular grafts. Additionally, the silk-based scaffold displayed promising hemocompatibility and biocompatibility in preliminary in vitro assessments [118].

Hybrid scaffolds, integrating ECM proteins with synthetic materials, have also been developed to enhance the biological activity of scaffolds for vascular tissue engineering. For instance, Goins et al. created non-thrombogenic inner lumens using a solid polymer film of poly(1,8 octanediol-co-citrate) (POC) and electrospun polymeric blends of POC, COL, and elastin as outer layers, aiming to leverage the inherent material properties for enhanced functionality [100,118].

In another work, Li et al. [119] proposed a dual–oriented/bilayered electrospun scaffold based on the use of a mixture of PCL, poly(D,L-lactide-co-glycolide) (PLGA), and Gt. An electrospinning system with a high-voltage power supply, a micropump, and a rotating stainless steel drum collector (diameter = 10 cm, speed 2500 r/m) was used to fabricate the scaffold. The resulting nanofibre sheet was then cut into a rectangle, with the fibres aligned along the longer side, and curled around a stainless-steel cylindrical roller (diameter = 5 mm). The junction was bonded with an aqueous solution of Gt. The outer layer was then electrospun onto the stainless-steel roller. This process resulted in a vascular scaffold with 7 cm length and 5 mm diameter. The morphology of all nanofibres obtained was fine and smooth and the diameter of the nanofibres in the inner and outer layers was 609 ± 251 nm and 657 ± 220 nm, respectively. In addition, the average pore sizes of the inner and outer layers were measured to be 55.2 ± 32.1 μm^2^ and 52.9 ± 35.3 μm^2^, respectively. The ultimate tensile stress of the dual-oriented/bilayered scaffold was 2.04 ± 0.15 MPa, while the elongation at break was 342.48 ± 4.13%. These values were also higher than those obtained for pure PLGA and Gt scaffolds. All these results indicated that the dual-oriented and bilayered scaffold had excellent mechanical properties, and the bursting pressure in the dry state was consistent with the systolic and diastolic pressure of a natural blood vessel. The results of the biological characterisation showed that the mixed scaffold had excellent biocompatibility and could promote the proliferation of both SMCs and ECs.

In addition to material selection, scaffold design plays a crucial role [100]. Simultaneous seeding of ECs and SMCs can mitigate thrombogenicity issues and aid in tissue remodelling. Structurally, scaffolds with porous outer layers for cellular infiltration and solid inner layers for EC attachment have shown promise in creating non-thrombogenic barriers [109]. Alternatively, scaffolds with porous outer and inner layers, resembling the arterial wall’s morphology and mechanics, have been fabricated to promote desirable EC and SMC functions, although these require additional steps to address thrombogenicity issues [110]. Fabrication techniques influence scaffold properties, with particulate leaching allowing for controlled pore size, while electrospinning produces nanosized pores resembling the arterial wall’s fibrous layers. Porosity size considerations are vital, varying by layer to accommodate cell size differences and prevent leakage or pressure drops during remodelling [120].

In the quest for fabricating biomimetic bilayered vascular scaffolds, researchers have explored innovative methods. First, Zhou et al. [116] employed an advanced coaxial 3D-bioplotter platform to create tunable vessels for small-diameter blood vessel replacements. Their method involved the simultaneous deposition of two types of bioinks containing methacrylated gelatin (GelMA), poly(ethylene glycol) diacrylate (PEGDA), alginate, and alginate lyase, cross-linked under UV light. VSMCs and vascular endothelial cells (VECs) were precisely seeded in the outer layer and lumen, respectively. Post-printing, vessels were cross-linked and the sacrificial inner core material was leached out, resulting in a hollow core structure. These vessels exhibited robustness, perfusability, and porous structures conducive to nutrient exchange and cell proliferation. Notably, VSMCs demonstrated uniform distribution and proliferation, with vessels containing alginate lyase showing faster proliferation. Viability assays confirmed high VSMC viability over time.

Meanwhile, Nguyen et al. [64] pioneered an electrochemical fabrication method to develop biomimetic bi-layered COL scaffolds incorporating insoluble elastin. The process involved creating a COL lumen via self-assembly triggered by an electric field and synthesising COL-elastin fibres through isoelectric focusing. Optimisation of electrochemical parameters ensured the formation of a compact, smooth, and bubble-free COL lumen, which is crucial for scaffold integrity. Mechanical testing revealed that circumferentially oriented COL-elastin fibres significantly enhanced scaffold mechanics, indicating their robustness and ability to withstand physiological forces. Furthermore, the scaffolds supported the expression of functional markers by ECs on the luminal surface, which is crucial for preventing thrombosis and promoting vascularisation.

**Figure 3:**
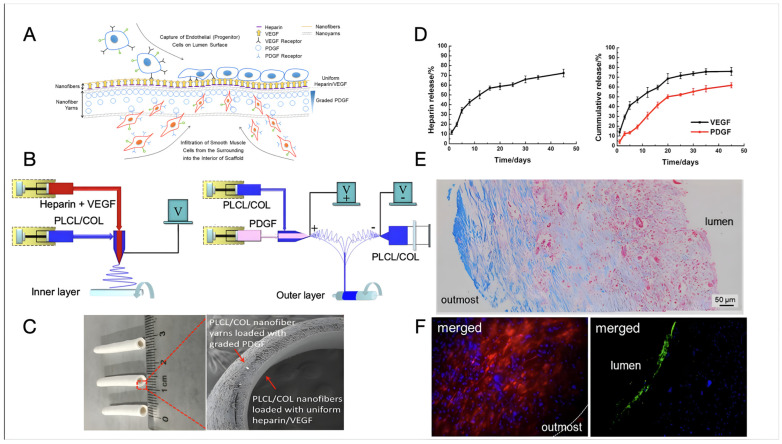
The design and fabrication of a bilayer vascular scaffold with spatially controlled release of growth factors from Wang et al. [121]. (**A**) A diagram depicting how the bilayer vascular scaffold should foster in situ tissue regeneration by capturing EPCs on the surface and allowing SMCs to infiltrate. Hep release is used to prevent early thrombus formation, while VEGF boosts endothelialisation and PDGF to facilitate SMC infiltration. (**B**) A schematic depiction of how the bilayer vascular scaffold was created using coaxial and conjugated electrospinning techniques. (**C**) A photograph displaying a sample of a vascular scaffold measuring 2 mm in inner diameter and 1 mm in thickness with SEM images illustrating its cross-section. (**D**) Graphs displaying the cumulative release of Hep (left), VEGF, and PDGF (right) from the vascular scaffold after incubation in a PBS solution on a horizontal shaker at 37 °C and 120 rpm for 45 days. (**E**) Microscopic images illustrating cross-sections of the bilayer vascular scaffold stained with Masson’s trichrome after being implanted in a rat abdominal aorta for two months. (**F**) Fluorescence micrographs depicting the vascular scaffold after immunohistochemical staining of DAPI and CD31 (lumen), and DAPI and α-SMA (outmost) following 2-month implantation in a rat abdominal aortic defect model. Open access article distributed under the CC BY-NC-ND license.

Recent examples of bi-layer design have led to some interesting results and significant advancements towards clinically relevant tissue-engineered vascular substitutes. Wang et al. [121] designed a bilayer vascular scaffold to facilitate in situ rapid endothelialisation and the alignment and infiltration of SMCs (Figure 3). The inner layer of the scaffold, made of electrospun poly(L-lactide-co-caprolactone) and COL (PLCL/COL) nanofibre, was loaded with Hep and vascular endothelial growth factors (VEGF), while the outer layer consisted of circumferentially aligned PLCL/COL nanofibre yarns loaded with platelet-derived growth factors (PDGFs) increasing in concentration from the outermost to the interior of the scaffold. The fabrication process involved coaxial electrospinning to create the inner layer and a modified setup integrating coaxial with conjugated electrospinning for the outer layer. The vascular scaffold was crosslinked using GTA vapours. The compliance of the vascular scaffold resulted comparable to that of a human saphenous vein (0.7–1.5%/100 mmHg) and superior to that of a commercial e-PTFE graft (0.1%/100 mmHg). The controlled release of VEGF and PDGF from the scaffold was investigated, with VEGF showing a continuous greater release percentage (ca. 15%) relative to PDGF over almost one month in vitro. The scaffold exhibited promising biological performance, with rapid endothelialisation observed at the luminal surface and oriented smooth muscles infiltrating inside the vascular wall at two months post-implantation in a rat abdominal aorta defect model. COL-rich connective tissues were produced at the outermost layer of the vascular scaffold, indicating its potential for in situ vascular repair or regeneration [121].

More recently, a scaffold reproducing the endothelial and media layers of blood vessels was developed by Ding et al. [122], using a combination of beta-sheet-rich silk nanofibers (BSN) and poly(vinyl alcohol) (PVA). BSN and PVA were blended in an aqueous solution and processed using a tubular electrode to form tubular scaffolds composed of circumferentially and axially oriented BSN near the positive electrode and non-crosslinked PVA. This step was followed by the crystallisation of PVA obtained by freeze–thawing, with the aim of providing mechanical strength to the construct without altering the aligned morphology of BSN. The obtained scaffold morphology effectively replicated the structural characteristics of the vascular intima and media layers. The crystallised PVA provided the scaffolds with suitable mechanical properties, including adequate suture retention strength. Compared to homogeneous scaffolds, the preferential alignment of smooth muscle and ECs induced by the developed scaffolds led to improved cell adhesion and proliferation, along with enhanced expression of various cytokines. In vivo studies demonstrated that endothelial layers form within four weeks of implantation, ensuring sustained patency. Furthermore, after eight months post-implantation, both endothelial and smooth muscle layers were regenerated, creating hierarchical microstructures and compositions that closely resemble native vessels.

While the bilayer approach offers benefits in terms of tissue organisation, culture time can be inhibitory, and addressing concerns regarding cellular infiltration and nutrient delivery to the scaffold central portion is essential. Additionally, comprehensive studies assessing mechanical properties, degradation, and in vivo performance are imperative for the clinical translation of scaffold-based vascular tissue engineering [109].

### 6.3. Three-Layer Scaffolds

**Figure 4:**
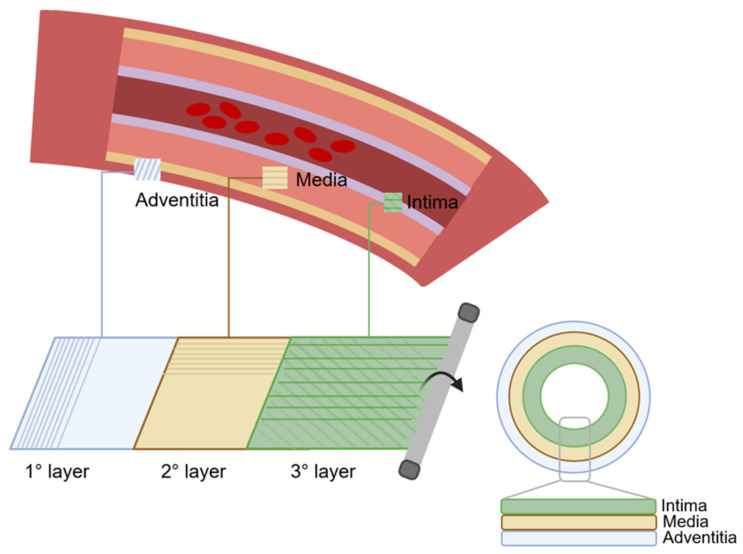
The preparation process of TEVGs using the three-layer technique. First, the outer layer is prepared, which corresponds to the tunica adventitia, then the second layer is prepared, which corresponds to the tunica media, and finally the third, which corresponds to the tunica intima. TEVG is obtained by rolling manipulation of the three layers.

Three-layer scaffolds represent the most closely biomimetic approach in vascular tissue engineering. The simulation of three-layer structures enables the acquisition of grafts that closely mimic the anatomy and dynamics of blood vessels (Figure 4). Consequently, there is an escalating inclination towards the investigation of tissue engineering methodologies aimed at replicating the three distinct anatomical layers [123,124].

Natural trilayer scaffolds, such as those utilising GelMA and alginate, have been developed through layer-by-layer deposition techniques [125]. Incorporating ECs within the intimal layer was shown to enhance endothelialisation, a crucial aspect of scaffold success. Similarly, hybrid scaffolds combining synthetic and natural materials have capitalised on the mechanical and biological advantages of each component. For instance, blending COL and elastin with synthetic polymers like PCL yielded compositionally mimetic layers with improved mechanical properties [126]. Research efforts have also explored various combinations of synthetic and natural polymers to fabricate tri-layer scaffolds with desirable mechanical and biological properties. These efforts include electrospinning techniques and the incorporation of COL, SF, or Ch into different scaffold layers [127]. In vivo assessments have demonstrated promising outcomes, including neovascularisation and cellular remodelling, suggesting the efficacy of COL-containing scaffolds in promoting regeneration [27].

The versatility of synthetic and natural polymers offers numerous possibilities for fabricating complex scaffold morphologies that mimic native ECM structures. However, the successful engineering of multi-layer scaffolds for small-diameter vessel applications necessitates careful consideration of design parameters and fabrication techniques, to ensure optimal performance and functionality.

Guo et al. proposed the construction of a biomimetic three-layer vascular scaffold with spatial alignment features, based on a blend of Gt, PCL, and PLGA [124]. A general strategy involving sequential electrospinning combined with folding and rolling manipulation was employed to achieve this. The resulting scaffold possessed three layers with inner and middle layers arranged perpendicular to each other, closely resembling the multi-layer structure of natural blood vessels. This architecture was branded essential to obtain integrated vascular remodelling and regeneration, facilitating spatial arrangement guidance for corresponding cells. Specifically, the inner layer promoted EC adhesion and proliferation, while the middle layer allowed for VSMC penetration. The outer layer provided mechanical support and biocompatibility. The sequential electrospinning process, combined with folding and rolling manipulations, ensured the formation of distinct layers with different fibrous arrangements and laminated structures.

Wu et al. [36] devised a novel tri-layer tubular graft composed of PLCL/COL fibres and PLGA/SF yarns using a three-step electrospinning approach. The graft had an inner layer of axially aligned PLCL/COL fibres, a middle layer of PLGA/SF yarns, and an outer layer of random PLCL/COL fibres. In the fabrication process, the axially aligned PLCL/COL fibres were first produced using a customised rotating collector under a magnetic field. Subsequently, PLGA/SF yarns were prepared via electrospinning with a double-nozzle system and twined onto the inner layer in a circumferential orientation. Finally, a layer of random PLCL/COL fibres was added on top to fix the entire structure in position. The obtained graft showed dense fibres on the inner and outer surfaces and a loose yarn structure in the middle layer. Mechanical testing revealed favourable tensile properties for each layer, with the inner layer providing structural support, the middle layer facilitating cell infiltration, and the outer layer fixing the yarns.

An important and often overlooked factor to consider when designing a blood vessel replacement is the discrepancy in compliance between the host vessel and the artificial vascular graft at the site of anastomosis. Compliance, in the context of vascular structures, refers to the dimensional alteration of a tube in response to changes in intraluminal pressure. This misalignment of compliance induces haemodynamic flow disturbances within the vascular graft and concentrates stress on the anastomotic junction. As a result, pressure changes at the anastomosis can lead to intimal hyperplasia and thrombosis [128,129]. To obtain grafts with adequate compliance, it is necessary to replicate the biomechanical characteristics of native blood vessels, and, in their study, Zhang et al. worked on making grafts that could meet this need [123]. They developed a three-layered vascular graft utilising a combination of electrospinning and braiding techniques, mimicking the structure of human blood vessels. The scaffold consisted of three distinct layers: intima, media, and adventitia. The intima and media layers were formed by electrospinning SF and PLCL, with varying ratios to match native tissue properties. The outer adventitia layer was braided using SF yarns to enhance durability against high pressures. Mechanical properties, including Young’s modulus and compliance, closely resembled those of natural blood vessels. Dynamic compliance values exhibited superior performance compared to synthetic Dacron grafts, with attained values indicating the compliance of the graft equivalent to that of the human saphenous vein. In vitro studies demonstrated cytocompatibility of the intima and media layers with ECs and SMCs, respectively.

The exploration of three-layered scaffolds for tissue engineering of blood vessels demonstrates significant promise in mimicking native vascular architecture; however, the current literature underscores not only the strengths but also the limitations. The versatility of these scaffolds allows for the replication of intricate vascular structures, fostering cellular infiltration and promoting tissue regeneration. However, challenges remain in achieving precise control over scaffold properties to replicate the dynamic mechanical behaviour and biochemical cues of natural vessels. Addressing these hurdles through continued research efforts holds the key to advancing the field of biomimetic scaffold architecture in vascular tissue engineering [9,27].

### 6.4. 3D Printed Scaffolds

**Figure 5:**
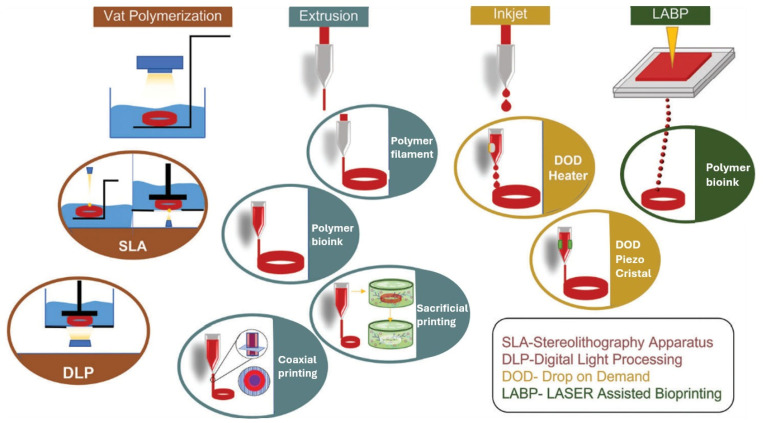
Different types of 3D bioprinting techniques employed for the generation of vascular grafts [130]. Open access article under the terms of the Creative Commons Attribution-NonCommercial License.

In recent years, 3D printing technology has revolutionised various fields, including tissue engineering. Specifically, 3D printing techniques enable the fabrication of complex structures using a range of polymers, including both synthetic and naturally derived materials. This technology has been instrumental in producing transplantable scaffolds and tissues, with inkjet, extrusion, and laser-assisted printing being the primary approaches used (Figure 5). Notably, 3D printing has facilitated the creation of SDVGs through innovative ink formulations and printing techniques [110].

Three-dimensional printing can produce two types of SDVGs: acellular or cellularised. Acellular SDVGs are vascular grafts that do not contain living cells, these grafts can serve as scaffolds for tissue regeneration, providing structural support and guiding the growth of new tissue. In contrast, cellularised SDVGs contain living cells within the scaffold structure and are produced by 3D bioprinting. Cellularised grafts have the potential to integrate seamlessly with patient tissues, promoting faster healing and potentially better long-term functionality [110]. Figure 5 shows the basic elements of the 3D printing technique for the fabrication of both acellular and cellularised SDVGs.

Using an advanced coaxial 3D bioplotter platform, Zhou et al. created biomimetic small-diameter blood vessels with two distinct cell layers [131]. A bioink made of VSMCs laden in GelMA/polyethylene(glycol)diacrylate and alginate lyase was used to fabricate the vessel wall, using a 3D extrusion-based bioprinter with a coaxial needle extrusion system. The presence of the coaxial needle enabled the simultaneous extrusion of two materials, one being the bioink and the other being Pluronic 127, which was used as a sacrificial material for supporting construction and then removed to generate a lumen structure. VECs were then seeded in the lumen, forming the inner layer of the vascular matrix. Since the space for the growth of the loaded cells was limited, lyase was introduced into the bioink to gradually degrade the alginate in the scaffold matrix. This special design favoured nutrient exchange with the environment while leaving more space for cell proliferation in the matrix. The obtained vessel exhibited significant perfusable properties under different conditions of flow velocity, flow viscosity, and temperature. The loaded VSMCs grew faster in the lyase group than in the group without lyase. After seeding the VECs, both the VSMCs in the matrix and the VECs in the lumen continued to grow steadily over time. This novel bioprinted blood vessel with biomimetic EC and SMC layers represents a potential candidate for the future replacement of small-diameter blood vessels.

**Figure 6:**
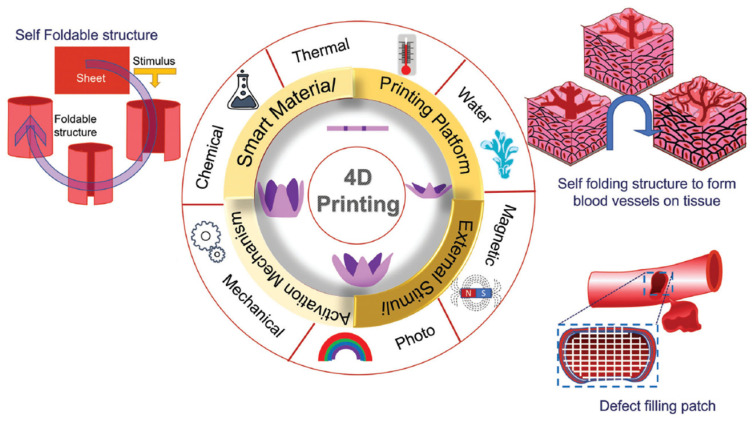
Main aspects of 4D printing and the application of 4D bioprinting in tissue engineering of blood vessels [130]. Open access article under the terms of the Creative Commons Attribution-NonCommercial License.

In addition to 3D printing, the emergence of 4D printing represents a significant advance in tissue engineering (Figure 6). In contrast to traditional 3D printing, a fourth dimension, time, is included, and it stems from materials that can transform in response to external stimuli such as pH, temperature, light, or humidity [110]. These “smart” materials dynamically change their shape and properties, enabling improved functionality in tissue engineering scaffolds [110]. Materials used in 4D printing include thermoresponsive polymers, pH-responsive polymers, and photoresponsive materials. Hydrogels are the predominant water-responsive material, while polyelectrolytes respond to electric fields, and magnetic nanoparticles respond to magnetic fields. Polymers such as COL and keratin respond to pH by changing the conformation of the polymer chain from a spherical to a coiled shape [130]. The potential applications of 4D printing are manifold. These include the development of vascular grafts that can adapt to changes in the body’s environment. A 4D-printed vascular graft could, for example, react to fluctuations in pH or temperature in the vascular system and possibly release therapeutic agents or adapt its shape accordingly. Although the field of “smart” materials in vascular engineering is still in its early stages, ongoing research ensures the development of advanced vascular grafts capable of recognising and responding to changes in the state of the human body [130].

## 7. Biomimicry in Scaffold Functionalisation

### 7.1. Surface Modification for Antithrombogenicity

The continuous endothelium of native blood vessels, composed of a monolayer of ECs, serves to prevent blood coagulation and regulate vascular tone through the synthesis and regulation of various bioactive molecules. Damage to the endothelium exposes blood to underlying ECM proteins, facilitating platelet adhesion and initiating the blood coagulation cascade. Therefore, incorporating natural anticoagulant molecules into TEVGs represents a fundamental strategy to mitigate acute thrombosis.

Notably, Hep, a naturally occurring polysaccharide, has been extensively utilised in clinical settings as an anticoagulant molecule. For example, Zhang et al. developed a three-layered vascular graft by electrospinning and functionalised the inner layer by Hep immobilisation through EDC/NHS chemistry [123]. The successful immobilisation of Hep on the scaffold was verified by toluidine blue staining. The hemocompatibility of heparinised scaffolds was then investigated by measurement of blood coagulation time, in vitro blood coagulation tests, and quantification of platelet adhesion. Prothrombin time and activated partial thromboplastin activity time were measured after incubation of both heparinised and non-heparinised scaffolds in human plasma solution, and the results were significantly higher for the heparinised ones. Additionally, Hep functionalisation of the scaffolds demonstrated enhanced anticoagulant properties following incubation in whole blood. Moreover, both platelet adhesion and platelet activation were reduced on the Hep-functionalised scaffolds.

Hep was also used to develop a biomimetic surface with anticoagulant properties to mitigate thrombus formation in the work published by Zhu et al. [117]. Poly(ester-urethane)urea/Gt nanofibrous tubular scaffolds were fabricated by electrospinning and then modified by Hep grafting through EDC/NHS chemistry. The resulting tubular scaffold exhibited improved blood compatibility in terms of both low hemolysis and low platelet adhesion. Additionally, the presence of Hep on the fibre surface enhanced EC proliferation at 7 days after seeding. In vivo studies further confirmed that the Hep-modified nanofibrous tubular scaffolds favoured long-term patency.

In another study, Gong et al. developed hybrid TEVGs by combining synthetic polymers with naturally decellularised small-diameter vessels [104,132]. The intimal surfaces of the hybrid SDVGs were coated with Hep before allograft transplantation to resist platelet aggregation and inhibit thrombosis. The effects of Hep functionalisation on platelet adhesion were investigated first by a platelet adhesion test with microscope observation and then by quantification of lactate dehydrogenase activity. Both tests showed that Hep modification significantly reduced the number of adhered platelets. Evaluation through vascular ultrasound and micro-CT angiography demonstrated satisfactory patency of all grafts implanted in a rat model for up to 6 weeks.

As Hep has a short in vivo half-life, researchers are looking into Hep-mimetic sulfated GAG and sulfonated polymers which are highly stable and possess Hep-like properties. In a work published by Kong et al. [113], nanofibres composed of a biomimetic blend of Gt and PCL incorporating varying amounts of CS were fabricated using electrospinning technology to examine their impact on antithrombogenicity and affinity towards ECs. In recent studies, CS was identified as a sulfated polysaccharide consisting of GAG and galactosamine. It was shown to exhibit strong adhesion to ECs while maintaining weak interactions with proteins and platelets, along with electrostatic repulsion of negatively charged blood components [133,134,135]. Moreover, CS was proven to inhibit cellular apoptosis and promote the healing process of vascular wounds [136,137]. The inclusion of different concentrations of CS in the nanofibres influenced their morphology and diameter. Moreover, the incorporation of CS into the nanofibres significantly improved their anticoagulant properties, extending their coagulation time, and promoting cell responses. Notably, nanofibres containing 10% CS demonstrated favourable outcomes in terms of cell attachment, elongation, and proliferation.

Alternatively, platelet adhesion can be avoided through the design of an anti-thrombogenic surface on the internal lumen of blood vessel scaffolds, replicating the continuous GAG layer present on ECs in native vessels. In this regard, a modification with hyaluronic acid on the internal surface of decellularised TEVGs has been investigated by Dimitrievska et al. to effectively shield blood platelets from activation triggered by COL [103]. By utilising the amine groups present on 4 mm diameter decellularised scaffolds a continuous hyaluronic acid hydrogel coating was constructed using a bifunctional thiol-reactive cross-linker, thus avoiding nonspecific COL matrix cross-linking. The hydrogel layer served to recreate a luminal wall, concealing exposed COL from direct contact with the bloodstream. In vitro blood tests demonstrated significantly lower levels of adhered platelets, fibrinogen absorption, and fibrin formation on the hyaluronic acid-coated decellularised scaffolds compared to the uncoated ones. Moreover, the hyaluronic acid surface exhibited inhibition of macrophage adhesion in vitro. Notably, in vivo experiments showed protection from aggressive thrombus formation, preservation of normal blood flow, and re-endothelialisation was also observed.

Another option to prevent acute thrombosis is represented by the immobilisation of NO. In native vessels, ECs are in contact with circulating blood and are subjected to continuous shear stress, which triggers the release of NO through the upregulation of endothelial NO synthase. This process results in vasodilation and suppresses platelet aggregation. To the best of our knowledge, however, this approach has so far been mainly investigated for vascular prostheses [138,139].

### 7.2. Surface Modification for Rapid Endothelialisation

The endothelium of native vessels plays a pivotal role in preventing thrombogenic complications and intimal hyperplasia, thus improving long-term patency. Therefore, although the incorporation of anti-thrombotic molecules onto vascular scaffolds plays a crucial role in preventing acute thrombosis, the sustained success of these grafts over the long term largely relies on the reconstruction of a continuous endothelium on the lumen surface.

Various methods have been investigated for in vitro endothelialisation, including the seeding with ECs on the inner surface of blood vessel scaffolds and subsequent in vitro maturation before implantation. These techniques have shown promise in enhancing graft patency; however, they also suffer from significant drawbacks. These include the need for supplementary procedures to harvest cells, particularly in the case of autologous cell use, risks of infections, prolonged culture times, and the associated high costs of the procedure [140].

Therefore, researchers’ attention is now mainly focused on in situ endothelialisation. Following implantation, endothelialisation of the graft typically occurs through one of three mechanisms: (i) transanastomotic growth, which involves the migration of host intimal ECs from the anastomotic site toward the centre of the graft, in response to natural injury-induced mechanisms; (ii) transmural growth, which occurs when ECs reach the lumen by traversing newly formed capillaries through the graft wall; (iii) fallout process, which entails circulating progenitor cells populating the implanted TEVG. Considering that, in humans, transanastomotic growth is limited to 1–2 cm [141] and the mechanism of transmural growth is not clear, the latter process involving circulating progenitor cells seems to be the most promising. EPCs are circulating blood cells able to differentiate into ECs and secrete multiple bioactive molecules [142]. Other less well-defined CD133^+^, CD34^+^, and CD45^−^ progenitor cells also hold the potential to be recruited onto a modified graft, facilitating in situ endothelialisation. Moreover, a specific subset of CD14^+^ monocytic cells was demonstrated to possess the ability to differentiate into ECs. Intriguingly, these monocyte lineage cells derived from the bone marrow, which are capable of maturing into ECs, have been reported to exhibit a more pronounced inhibition of intimal hyperplasia compared to EPCs [143]. Therefore, various strategies have been developed to promote rapid in situ endothelialisation through the modification of the scaffold lumen with biomolecules promoting circulating progenitor cell migration, adhesion, proliferation, and differentiation.

Considering that EPCs express specific markers, such as CD133, CD34, and the kinase domain receptor, along with co-expressing markers indicative of their hematopoietic origin, specific antibodies targeting these markers have been used for scaffold surface functionalisation to facilitate the capture of EPCs [144]. For example, Wu et al. developed a bi-layer scaffold by electrospinning, which included the luminal and media layers. The luminal layer was loaded with Hep and anti-CD133 antibody [35]. The loaded bioactive components were gradually released over an approximately 40-day period, during which the structure of the nanofibres remained intact. Additionally, the released Hep initially provided anticoagulant functionality of the lumen, while the incorporated anti-CD133 antibody facilitated the formation of a neo-intima. In vivo tests revealed the regeneration of a monolayer of ECs (positive for CD31) on the inner layer, demonstrating the scaffold’s capability to regenerate structures resembling native blood vessels. Nonetheless, despite demonstrating promising endothelialisation, grafts coated with antibodies led to excessive intimal hyperplasia at the anastomoses and markedly increased platelet adhesion [145,146].

Alternatively, VEGF and numerous other bioactive factors, such as interleukin-8, stromal cell-derived factor-1 (SDF-1), angiopoietin-1, and basic fibroblast growth factor, have been used to favour ECs proliferation and survival. In the work published by Hu et al., small-diameter tubular scaffolds were fabricated using the coaxial-electrospinning method and the fibre core was loaded with Hep and VEGF [147]. A gradual release of Hep and VEGF was registered over 28 days. In vitro and in vivo tests demonstrated an improvement in terms of EC proliferation and patency with respect to non-functionalised grafts. Similarly, Du et al. developed a gradient heparinised nanofibrous scaffold to support ECs lining on the inner surface [148]. To mimic the natural environment of blood vessels, Hep was covalently attached to the scaffold by EDC/NHS chemistry and VEGF was loaded in the nanofibres by adsorption. The amount of heparinised nanofibres gradually increased from the outer layer (tunica adventitia) to the inner surface (lumen) of the scaffold. Enhanced anti-thrombogenic properties of the heparinised scaffolds were demonstrated by activated partial thromboplastin time and platelet adhesion assays. Furthermore, the release of VEGF was stable and sustained, with a lower burst release within the initial 12 h. EC adhesion and proliferation were significantly improved, and the formation of a complete monolayer was observed.

Another strategy to promote rapid endothelialisation is represented by scaffold functionalisation with bioactive peptides derived from ECM components. It was demonstrated that several peptide sequences can be bound by receptors on cell membranes and consequently influence cell behaviour. The most investigated peptide sequence for scaffold modification is Arg-Gly-Asp (RGD) from fibronectin. However, as the RGD sequence is recognised by receptors that are present in all cell types, it may induce not only EC adhesion but also adverse cell adhesion and platelet aggregation. It has been reported in the literature that ECs exhibit a higher affinity for immobilised cyclic RGD peptides compared to linear RGD peptides [149]. Remarkably, aside from promoting EC adhesion, certain cyclic RGD peptides have demonstrated potent and selective antagonistic effects on the platelet integrin α2β3, effectively inhibiting platelet-mediated thrombus formation [150]. It is worth noting that ECs express a diverse range of integrins, with at least 13 variants identified thus far, depending on their developmental stage, differentiation state, and functional context. Notably, α4 integrins, which are absent in platelets, represent an attractive target for ligands aimed at facilitating in situ endothelialisation of vascular grafts [151]. For example, the tetrapeptide REDV, derived from fibronectin, exhibits a specific binding affinity for the α4β1 integrin. As the α4β1 integrin is predominantly expressed in ECs and EPCs, REDV possesses the ability to selectively enhance the adhesion of ECs/EPCs [152,153].

In an interesting paper, published by Duan et al., a combinatorial approach was explored, involving the successful co-immobilisation of the REDV peptide, VEGF, and CD133 antibodies onto a blood vessel scaffold [154]. This modified surface exhibited favourable compatibility with blood and notably increased its capacity to capture CD133^+^ EPCs. The co-immobilisation of REDV and VEGF significantly enhanced the proliferation of EPCs. Furthermore, it conspicuously elevated the expression of EC marker genes in EPCs, thereby promoting the differentiation of EPCs into ECs.

In the paper published by Rosellini et al., tubular scaffolds based on a protein/polysaccharide blend were functionalised using two different bioactive peptides, Gly-Arg-Gly-Asp-Ser-Pro (GRGDSP) and Arg-Glu-Asp-Val (REDV) [82]. In vitro cell culture tests showed that the functionalisation with the REDV peptide favoured the adhesion and growth of ECs, and therefore could be advantageous for the endothelialisation of the internal surface of an engineered vessel, while GRGDSP promoted the adhesion of fibroblasts, which are present in the external layer of the vessel wall. Therefore, a bi-functionalised tubular scaffold could be developed, with REDV grafted on the luminal side of the scaffold, to specifically promote EC adhesion, and GRGDSP immobilised on the external side, to promote fibroblasts and SMC adhesion. This bi-functionalised scaffold could be able to promote the regeneration of a layered tissue, resembling the native structure of small-calibre arteries.

Besides biochemical modifications of scaffolds for TEVGs, the geometrical configuration of the surface also plays a role in the in situ endothelialisation of grafts [155]. This is because the nanofibrous ECM found in native vessels provides numerous physical and biological cues that regulate homeostasis, morphogenesis, and endothelial cellular functions [156]. Therefore, in recent years there has been a growing interest in exploring the impact of interfacial properties, including surface micro/nano topography, on cell behaviour. For example, it was demonstrated that nano-scale fibres, replicating the dimensions of the natural ECM, are conducive to the adhesion and proliferation of ECs [157,158]. In another work, it was shown that EC coverage was promoted on nanometer-scale patterns, instead of micrometre-scale patterns [159]. Not only the presence of nanostructures on scaffold surfaces but also their orientation has an impact on cell response. In a concerted approach, reported by Wang et al., a biomimetic intima featuring an oriented nanotopographical structure and covalently immobilised anticoagulant molecules was investigated [160]. A blend of heparinised SF and PCL was utilised to create the oriented inner layer of the scaffold. The obtained results demonstrated that the immobilised Hep significantly influenced platelet adherence and activation, whereas the oriented nanotopography primarily influenced the elongation and alignment ofECs, as well as the hemodynamics of blood flow. Notably, the synergistic effects of the oriented structure and anticoagulation significantly promoted rapid endothelialisation, long-term patency, and neovessel remodelling. Consequently, this study successfully combined biochemical induction via Hep molecules with biophysical stimulation through oriented nanotopography to develop an off-the-shelf SVDG with excellent early-stage antithrombotic properties and sustained patency in the long term. In another paper, the authors focused on the biomimetic design of an acellular SVDG, featuring a specific lamellar nanotopography on the luminal surface achieved through a modified freeze-cast technique. Experimental findings confirmed that the nanolamellar structure effectively impeded platelet adherence and activation, promoted the oriented growth of ECs, and ultimately facilitated the remodelling of neovessels to sustain long-term patency in vivo. Additionally, numerical simulations conducted under physically mimetic conditions demonstrated that the regular lamellar nanopattern could manipulate blood flow to decrease flow disturbance compared to random topography.

### 7.3. Scaffold Functionalisation for Preventing Intimal Hyperplasia

Intimal hyperplasia is a pathological condition characterised by the thickening of the vessel wall, stemming from the excessive growth and migration of SMCs from the medial layer into the intimal layer due to endothelial damage [161]. In a healthy blood vessel, the endothelium regulates the underlying SMCs by inhibiting their proliferation and promoting a quiescent, differentiated state characterised by a contractile phenotype [162]. Any unintended damage to the endothelium exposes the medial layer, leading to platelet activation. This triggers a transition in the phenotype of SMCs from a quiescent, contractile state to a highly proliferative one, ultimately resulting in intimal thickening. Surgical implantation of vascular grafts typically damages the endothelium, exposing the medial layer and thereby increasing the risk of developing intimal hyperplasia. Techniques aimed at inducing rapid endothelialisation may not suffice to prevent the thickening of the graft wall. The biological and mechanical properties of TEVGs serve as critical regulators of hyperplasia development.

The strategies employed to prevent intimal hyperplasia in bioengineered vascular grafts predominantly revolve around the incorporation of drug molecules aimed at reducing SMC proliferation and platelet adhesion. Potential drugs targeted for this purpose include sirolimus or rapamycin [163], aspirin [164], and paclitaxel [165]. However, the burst release of the drug, propelled by blood flow, minimises the interaction between cells and the drug, thereby challenging the overarching concept. Moreover, antiproliferative drugs may result in delayed endothelialisation and cellular ingrowth, leading to a reduction in neointima formation. The delayed endothelial coverage may pose an issue regarding early thrombogenicity.

Knowing these limitations, an alternative strategy was developed by Ding et al., who proposed an irregular mesh made of carbon nanotubes, and loaded with resveratrol, as a coating on the scaffold lumen [166]. The mesh aimed to slow down the drug release caused by blood flow, thanks to a high resistance against shear stress. Resveratrol was internalised by proinflammatory M1 macrophages and caused their conversion to the pro-healing M2 phenotype. The pro-healing M2 macrophages play a pivotal role in inhibiting chronic inflammation, thereby preserving the contractile phenotype of VSMCs, consequently mitigating intimal hyperplasia. Furthermore, the release of resveratrol from the mesh coating directly protected contractile VSMCs from transitioning to a secretory phenotype. By employing an anti-shear stress coating and macrophage-based intracellular drug delivery, the developed tissue-engineered blood vessel demonstrated sustained efficacy against intimal hyperplasia over the long term. Animal transplantation studies conducted on rat carotid arteries revealed a consistently high patency rate up to day 90 post-grafting.

A different biologically inspired approach was followed by Sugiura et al., who investigated the efficacy of tropoelastin seeding onto the luminal surface of the graft in preventing neointimal hyperplasia [167]. Tropoelastin was effective in reducing the thickness of the intimal layer, suppressing neointimal SMC proliferation.

Therapeutic strategies targeting both proliferation and inflammation have demonstrated efficacy in preventing intimal hyperplasia in both natural and synthetic blood vessel grafts. However, it is surprising that, despite being a fundamental factor in the development of intimal hyperplasia, the compliance mismatch between native vessels and TEVGs is often overlooked, with hypo-compliant (stiffer) grafts commonly utilised. In a recent paper, the authors aimed to address this issue by producing a vascular substitute that matches the compliance of the abdominal rat aorta through computational optimisation [168]. One-month follow-up observations revealed consistent graft compliance and an increased expression of markers associated with contractile SMCs in the compliance-matched group.

### 7.4. Strategies to Control Cell and ECM Arrangement

As depicted in Section 2, blood vessels possess a structural complexity that serves to maintain tissue balance by overseeing biological and physical attributes. Within the blood vessel, the luminal layer (intima) comprises a monolayer of ECs aligned with the blood flow direction, while the medial layer consists of radially aligned SMCs, and the adventitia contains randomly oriented fibroblasts. Moreover, COL and elastin fibres in the arterial wall are aligned at specific angles, influencing mechanical properties. Manipulating cell function through engineering at the cell-substrate interface presents promising avenues. A recent study demonstrated that surface properties, such as wettability, roughness, patterning, and stiffness, regulate the functionality of vascular cells [169].

The manipulation of nanoscale-oriented geometry of the intimal layer was demonstrated to have a regulatory effect on the hemocompatibility of grafts and to facilitate in situ endothelialisation [140,170]. Similarly, inducing unidirectional alignment of SMCs cultured on micro-patterned substrates has been observed to promote a transition from a proliferative to a contractile phenotype [171], potentially mitigating the risk of intimal hyperplasia. In another work, inspired by the radial arrangement of SMCs in native vessels, tubular scaffolds incorporating circumferentially oriented nanofibres were fabricated using electrospinning to facilitate the unidirectional alignment of SMCs via contact guidance [172]. In an analogous endeavour, a dual-layered tubular graft containing microfibers circumferentially aligned in the layer replacing the media attracted radially organised host SMCs after implantation into rat abdominal aortas. Histological examination indicated the absence of hyperplasia, with the regenerated neovessel demonstrating responsiveness to vasoactive agents [173]. Overall, these findings underscore the promise of engineering biomimetic grafts that mimic the native cellular organisation in blood vessel layers: the circumferential alignment of medial SMCs enhances mechanical properties and prevents hyperplasia by limiting cellular overgrowth and the nanolamellar luminal topography targets the orchestration of an anti-thrombotic surface, thereby inhibiting platelet adhesion.

While achieving native-like orientation of vascular cells has shown promising results, there is also significant research interest in exploring other structural aspects, as native tissues contain cells and an ECM. As mentioned above, one trending prospect in this field is the development of multilayered TEVGs. These systems aim to mimic the complex wall structure of natural vessels, which consist of multiple layers of different cell types and ECM components. By replicating this multilayered structure, researchers hope to enhance the performance and functionality of the grafts, potentially leading to improved outcomes in vascular tissue engineering, as described in Section 6. Moreover, the regeneration of ECM components is closely associated with the infiltration of fibroblasts and SMCs. Fibroblasts primarily contribute to the regeneration of COL but have a limited impact on other ECM components such as elastin and GAGs; on the other hand, SMCs play a crucial role in fostering the ECM [174,175]. Researchers have conducted numerous studies investigating the impact of graft microstructure on ECM regeneration. Initially, porosity and pore size were highlighted as critical factors influencing cell infiltration. Subsequent investigations revealed a strong correlation between the regeneration of SMCs and high porosity/pore size. Conversely, the structural requirements for endothelial cell adhesion and proliferation differed significantly. Consequently, developing multi-layered scaffolds, which can accommodate the distinct requirements of both ECs and SMCs, seems to be a promising strategy. Additionally, previous research has demonstrated that certain growth factors can facilitate ECM regeneration. Specifically, PDGF has been identified as capable of attracting SMCs and pericytes, while transforming growth factor-beta is deemed crucial for ECM deposition. Furthermore, DKK-3 has been observed to play a role in enhancing the maturation of SMCs [174,176].

### 7.5. Tailoring Immune Response

Immunoengineering represents a recent advancement within the field of tissue engineering, focusing on directing the immune system towards facilitating tissue regeneration. Recognition of the pivotal role of the host immune response in guiding the remodelling of TEVGs emerged following notable research efforts of Roh et al., who demonstrated the involvement of inflammation in graft maturation [177]. Subsequent investigations also underscored the critical regulation of stenosis by the innate immune system [178]. The degree of inflammation governs the formation of new tissue, with excessive inflammation leading to graft stenosis. While ongoing research continues to identify key immune system components involved in vascular graft remodelling, macrophages have emerged as primary regulators [179]. These cells, along with fibroblasts, play a crucial role in determining biomaterial-induced fibrosis and the formation of new ECM [180]. The intricate balance between proinflammatory M1 and various anti-inflammatory M2 macrophage subtypes orchestrates the overall remodelling process. Macrophage plasticity offers a significant opportunity for tissue engineers to influence their function towards promoting favourable graft remodelling outcomes.

As recently reviewed by Zhang et al., numerous studies have highlighted the impact of biomaterial properties on macrophage response and, consequently, on vascular tissue regeneration [181].

Fiber size and pore size represent two physically controllable factors that significantly influence macrophage response. Wang et al. conducted a study where they fabricated electrospun PCL vascular grafts with varying fibre thickness and average pore size [182]. They found that grafts with thicker fibres and larger average pore sizes facilitated a moderate level of macrophage infiltration and induced a stronger M2 macrophage response. Conversely, substrates with thinner fibres and smaller average pore sizes limited macrophage infiltration and predominantly elicited an M1 macrophage response. Moreover, grafts with thick fibres and large average pore sizes exhibited superior patency in a rat abdominal aortic model for a duration of up to 100 days. In the study, the authors also noted the presence of M1 macrophages throughout the entire observation period, while M2 macrophages were observed as early as Day 7 post-implantation, and persisted for 100 days. In line with these findings, Liu et al. documented that nanofibre vascular grafts prompt a more intense inflammatory response and calcification in comparison to microfibre grafts. They observed that macrophage infiltration and polarisation towards a pro-regenerative phenotype were augmented by grafts with thicker fibres and larger pore sizes, particularly on the circumferential outer side of the graft [183]. Other studies investigated the effect of micro/nanotopography, mechanical properties (in terms of stiffness and radial compliance), and chemical composition on macrophages; however, the impact of these scaffold properties on macrophage phenotype polarisation was not completely clarified [181].

As an alternative to the customisation of scaffold properties, the immobilisation or encapsulation of signalling molecules on the surface or within the scaffold to achieve sustained release is now recognised as an additional strategy to modulate the immune response and enhance tissue regeneration. For example, acellular TEVGs functionalised with VEGF were found to have a greater abundance of M2 macrophages [184]. Conversely, grafts solely functionalised with Hep predominantly supported M1 macrophages and exhibited a limited development of vascular endothelium and medial layers. In another work, Bonito et al. designed a PCL scaffold conjugated with Hep and IL-4 which effectively stimulated a high ratio of M2 to M1 macrophages in vitro [185]. Recently, Wei et al. devised a method for releasing mesenchymal stem cell-derived small extracellular vesicles from a heparinised vascular graft to bolster vascular regeneration [186]. Their observations revealed that grafts loaded with sEVs exhibited fewer M1 macrophages and a higher proportion of M2 macrophages. In a separate cell culture investigation, grafts laden with sEVs were capable of inducing macrophage polarisation towards the M2 phenotype at the gene expression level. The prevalence of the M2 macrophage phenotype was associated with superior vascular outcomes, such as enhanced patency rates and the regeneration of continuous endothelium and contractile SMCs within the neotissue.

## 8. Biomimicry in Bioreactor Design for Tissue Maturation

Notoriously, creating a three-layer vascular substitute—containing ECs, SMCs, and fibroblasts—is insufficient to generate a functional blood vessel. The subsequent phase, known as maturation, is complex, crucial, and time-consuming. During maturation, cells adapt to their 3D environment, produce an ECM, and establish cell–cell connections. Achieving tissue-like maturation requires an in vivo-like environment, making bioreactors crucial in vascular tissue engineering. Initially focused on endothelialising scaffold layers, bioreactors now play a vital role in controlling cell maturation and proliferation within constructs [9,187,188]. Bioreactors are essential tools in tissue engineering, designed to recreate a variable degree of the natural conditions for cell growth and tissue formation. Effective bioreactor design incorporates biomimetic principles to enhance tissue functionality by applying physiological conditions to stimulate cellular responses [189,190]. The tissue maturation process comprises an initial post-production stage, after which the tissue is generally fragile and unstable and necessitates measures to ensure cell survival and attachment to scaffolds or substrates. Over the subsequent weeks, tissue development and maturation occur. During this period, the bioreactor should offer dynamic conditions and customised stimulation to optimise tissue response while progressing its functionality and integration with host tissue. Gradually, the tissue may be exposed to challenging conditions in the bioreactor, including mechanical stimulation. Evaluation of tissue performance and quality remains critical throughout this phase [191,192].

In the field of blood vessel tissue engineering, bioreactors are categorised into three types: static, dynamic, and biomimetic. Static bioreactors passively mature constructs in standard vessels within controlled atmosphere incubators. Dynamic bioreactors consist of vessels with inlet and outlet, perfusing ECs for endothelialisation or inducing cell maturation through hydrodynamic stress. Biomimetic bioreactors aim to recreate in vivo tissue environments, controlling parameters like perfusion flows, pH, temperature, oxygen, and nutrient supply. Moreover, they aim to provide optimal conditions for cell proliferation and viability, mimicking physiological conditions, such as shear stress, to favour the development of vascular cell functions [9,188,193]. Researchers have utilised various commercially available bioreactor systems to replicate physiological vascular conditions, which implement pulsatile pumps, oscillators, culture medium reservoirs, and advanced scaffold chambers [194,195]. Nonetheless, homemade bioreactors are prevalent in vascular tissue engineering. Bono et al. developed a dual-mode bioreactor for construct fabrication and in vitro stimulation. At the same time, Iris Pennings et al. created a homemade perfused bioreactor for a bi-layered vascular graft, applying physiological shear rates to the luminal side for EC proliferation and exposing the outer layer to static culture conditions [196,197].

In vascular tissue engineering, the choice of a bioreactor system depends on the desired maturation conditions of the vascular construct. Various maturation strategies exist, including flow through the vessel, mechanical stimulation with a rotational bioreactor, or burst pressure. Studies have shown the crucial role of shear stress in cell migration and proliferation. Bioreactors can simulate shear stress through flow, pulses, or mimicking heartbeat [9]. For instance, a study by Chen Wang et al. demonstrated significant improvements in the biomechanical properties of vascular constructs under dynamic stimulation compared to static conditions. However, current bioreactors mostly focus on obtaining endothelialisation, leaving gaps in the maturation of other vascular layers [198,199].

**Figure 7:**
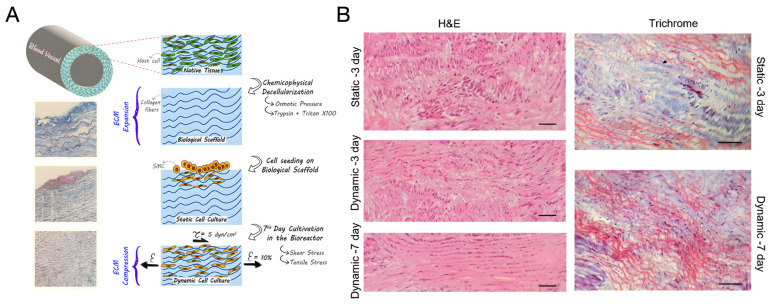
The attempt of Omid’s group [101] the quality of bioreactor-mediated tissue construct maturation. (**A**) A graphical scheme illustrating their novel decellularisation method for biological scaffolds. Chemical and physical techniques, including hypertonic/hypotonic solutions and trypsin/Triton X-100 treatment, were employed to remove cellular components while preserving ECM integrity. Conducting decellularisation at 4 °C minimised damage, maintaining mechanical strength, and facilitating optimal cell attachment. Biocompatibility was confirmed via MTT assay, with storage viability at −20 °C. (**B**) Histological images of the biological scaffold following 3-day static, 3-day dynamic, and 7-day dynamic SMC cultivation, stained with H&E and Masson’s trichrome, reveal distinct structural changes. Dynamic culture resulted in aligned and compressed COL filaments, facilitating cellular penetration (scale bar = 300 μm). The article is licensed under a Creative Commons Attribution 4.0 International License.

Despite advancements like 3D bioprinting, newly created vascular tissues lack sufficient mechanical properties and require maturation in a bioreactor. This results in the known paradox for which immature blood vessels need a bioreactor for functional maturation, but they are too fragile to be placed in one [9]. Nonetheless, there have been a consistent number of attempts at improving the quality of bioreactor-mediated tissue construct maturation by addressing the current limitations of this practice. Omid et al., for instance, addressed the limitations of traditional decellularisation methods, which often result in damage to ECM proteins and functional peptides, hindering the quality of biological scaffolds for vascular tissue engineering (Figure 7). By employing a combination of chemical and physical decellularisation methods and utilising a bioreactor for cyclic tensile and shear stresses, the researchers sought to enhance cell migration into the scaffold and improve the compactness of cells and scaffold, mimicking native tissue structure [101].

Derhambaksh et al. sought to tackle a key obstacle in vascular tissue engineering particularly concerning small-diameter vessels: the aberrant proliferation and migration of VSMCs from the media layer of the artery. By converting VSMCs from a synthetic to a contractile phenotype using electrical stimulation, the researchers aimed to prevent abnormal growth and migration, thus addressing a critical challenge in achieving successful vascular grafts [200]. On another note, Kojima et al. addressed the need for biologically compatible vascular grafts by exploring scaffoldless tissue engineering methods for fabricating functional vascular mimetics. By utilising hydrostatic pressurisation under hypoxia, the researchers aimed to promote the formation of multi-layered tunica media from human VSMCs, offering a potential solution for producing scaffoldless human vascular grafts [201].

## 9. Concluding Remarks and Future Directions

Blood vessel engineering represents a critical frontier in addressing the challenges posed by CVDs, notably atherosclerosis. The intricate nature of healthy arteries underscores the complexity inherent in replicating their functionality through tissue engineering. Although the fabrication of functional vascular substitutes is still a challenge, an incredible amount of research activity has been carried out in this field and significant advancements in scaffold-based methodologies offer promising avenues.

As illustrated in this review, the engineering of small-diameter blood vessels able to recapitulate all the biological and mechanical properties of native vessels requires the selection and combination of appropriate strategies for scaffold development. In particular, the need has arisen to follow a biomimetic approach on multiple fronts, including the choice of materials, the scaffold architecture, the functionalisation strategies to enrich the scaffold with signals, and the design of a dynamic culture environment (bioreactor).

The exploration of various scaffold materials and fabrication techniques reveals a concerted effort to mimic the native features of arteries. Natural polymers, such as COL, Gt, fibrin, and elastin, along with decellularised tissues, provide biocompatible substrates that closely resemble the composition of native ECM, offering superior cell response. Strategies to optimise scaffold architecture, mimicking the multi-layer structure of native vessels, are crucial for achieving long-term functionality and patency rates comparable to natural vessels. In this regard, electrospinning and, more recently, 3D printing, have emerged as the most promising techniques for this purpose, enabling precise control over scaffold morphology and bioactive component distribution, facilitating cellular organisation and tissue maturation.

Innovations in scaffold design extend beyond mimicking compositional and structural features to encompass functional aspects that are crucial for clinical success. Surface modification plays a pivotal role in enhancing hemocompatibility, promoting endothelialisation, and mitigating thrombosis risk. Functionalisation strategies can also enrich the scaffold with biochemical and topographical signals, which are useful to prevent intimal hyperplasia, guide cell and ECM organisation, and tailor the immune response. All of these aspects are crucial to obtain fully functional vascular grafts.

Moreover, the maturation of functional vascular tissue on the biomimetic scaffolds should be further promoted through the development of bioreactors for cell culture replicating the native biomechanical environment of small-diameter blood vessels.

Looking ahead, the field of vascular tissue engineering requires further advancements driven by ongoing research efforts and technological innovations. Currently, no biomimetic multi-layer vascular graft has reached clinical application. As outlined in this review, a multi-faceted biomimetic approach in scaffold design seems to be the right path towards clinical translation. At the same time, addressing challenges such as batch-to-batch variability, and the need to find a balance between scaffold complexity and scalability, will be paramount for translating experimental findings into clinically viable solutions. Ultimately, the convergence of interdisciplinary expertise and collaborative endeavours will be instrumental in realising the full potential of scaffold-based approaches for vascular repair and regeneration.

## Data Availability

No new data were created or analysed in this study. Data sharing does not apply to this article.
